# Hypochlorous acid-modified human serum albumin suppresses MHC class II - dependent antigen presentation in pro-inflammatory macrophages

**DOI:** 10.1016/j.redox.2021.101981

**Published:** 2021-04-20

**Authors:** Agnes Ulfig, Verian Bader, Marharyta Varatnitskaya, Natalie Lupilov, Konstanze F. Winklhofer, Lars I. Leichert

**Affiliations:** a Ruhr University Bochum, Institute of Biochemistry and Pathobiochemistry – Microbial Biochemistry, Universitätsstrasse 150, 44780, Bochum, Germany; b Ruhr University Bochum, Institute of Biochemistry and Pathobiochemistry – Molecular Cell Biology, Universitätsstrasse 150, 44780, Bochum, Germany

**Keywords:** Macrophages, Antigen presenting cells, Hypochlorous acid, N-chlorination, MHC II, CD36

## Abstract

Macrophages are innate immune cells that internalize and present exogenous antigens to T cells via MHC class II proteins. They operate at sites of infection in a highly inflammatory environment, generated in part by reactive oxygen species, in particular the strong oxidant hypochlorous acid (HOCl) produced in the neutrophil respiratory burst. HOCl effectively kills a broad range of pathogens but can also contribute to host tissue damage at sites of inflammation. To prevent tissue injury, HOCl is scavenged by human serum albumin (HSA) and other plasma proteins in interstitial fluids, leading to the formation of variously modified advanced oxidation products (AOPPs) with pro-inflammatory properties. Previously, we showed that HOCl-mediated N-chlorination converts HSA and other plasma proteins into efficient activators of the phagocyte respiratory burst, but the role of these AOPPs in antigen presentation by macrophages remained unclear. Here, we show that physiologically relevant amounts of N-chlorinated HSA can strongly impair the capacity of THP-1-derived macrophages to present antigens to antigen-specific T cells via MHC class II proteins at multiple stages. Initially, N-chlorinated HSA inhibits antigen internalization by converting antigens into scavenger receptor (SR) ligands and competing with the modified antigens for binding to SR CD36. Later steps of antigen presentation, such as intracellular antigen processing and MHC class II expression are negatively affected, as well. We propose that impaired processing of pathogens or exogenous antigens by immune cells at an initial stage of infection prevents antigen presentation in an environment potentially hostile to cells of the adaptive immune response, possibly shifting it towards locations removed from the actual insult, like the lymph nodes. On the flip side, excessive retardation or complete inhibition of antigen presentation by N-chlorinated plasma proteins could contribute to chronic infection and inflammation.

## Graphical abstract

**Figure:**
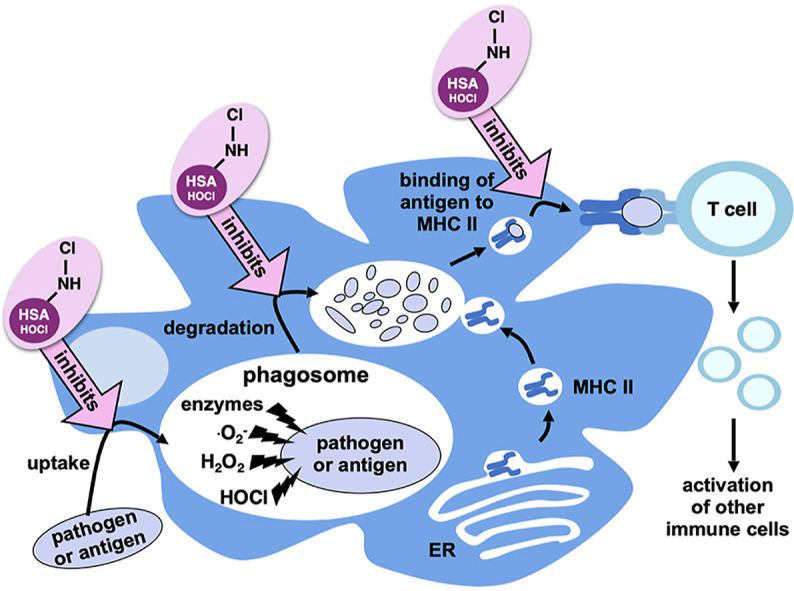

## 1 Introduction

Blood monocytes and their macrophage descendants are vital for the defense against pathogens due to their role in antigen presentation, and for the maintenance of tissue and organismal homeostasis [[1], [2], [3]]. Presentation of antigens by macrophages to CD4^+^-T helper cells via MHC class II molecules (HLA-DP, -DQ, and -DR) is critical for initiating the adaptive immune response to defend against infection (reviewed in Refs. [4,5]). Antigen presentation by macrophages is a multistep process involving (1) recognition and internalization of a pathogen or exogenous antigen (Ag), (2) pathogen/Ag degradation in endo/lysosomal compartments by proteases, (3) synthesis of MHC class II proteins in the endoplasmic reticulum (ER) and their vesicular transport to the antigen-containing compartments, (4) loading of pathogen/Ag-derived peptides on MHC class II molecules, (5) migration of the MHC class II-peptide complex to the cell surface, and finally (6) presentation of the bound peptides to peptide-specific CD4^+^-T cells through interaction of the MHC class II complex with T cell receptors (TCRs) [[4], [5], [6], [7]]. This interaction activates the T cell, which can then proceed with initiation of the adaptive immune response by secreting cytokines to propagate inflammation and activating B cells to produce antibodies [8].

Another important type of white blood cells, the neutrophils, provide the first line of defense against invading pathogens and are the major source for reactive oxygen species (ROS) and the heme enzyme myeloperoxidase (MPO), which generates the powerful oxidant hypochlorous acid (HOCl) in the presence of hydrogen peroxide (H_2_O_2_) and chloride ions (Cl^−^) [[9], [10], [11]]. Although MPO and HOCl exhibit strong antimicrobial activity and effectively kill invading pathogens, their extracellular secretion at sites of inflammation also places the human body at considerable risk [[12], [13], [14], [15], [16]]. HOCl reacts instantly with a wide range of biomolecules, such as proteins [14,[17], [18], [19]], DNA [20], cholesterol [21], and other lipids [22] present in its immediate vicinity to form oxidized and chlorinated products.

Owing to their high abundance in circulation and interstitial fluids, however, human serum albumin (HSA) and other plasma proteins were found to be the major target for HOCl and as such, represent the main scavenger of HOCl at sites of infection and inflammation [17,18,[23], [24], [25]]. Exposure of HSA and other plasma proteins to HOCl is known to result in the alteration of their amino acid side chains. Modifications that have been observed on proteins under these conditions include reversible and irreversible oxidation of sulfur-containing amino acids (i.e. cysteine and methionine), and the reversible N-chlorination of basic amino acids [26,27]. Other oxidative modifications lead to the chlorination of arginine and tyrosine side chains, oxidation of tryptophane and histidine residues [18,19,24,[28], [29], [30], [31]], or intermolecular di-tyrosine cross-linking [32]. This can eventually lead to irreversible fragmentation [33,34] and misfolding/aggregation [35]. The resulting oxidation products, termed “advanced oxidation protein products” (AOPPs) [36,37], commonly accumulate in many chronic inflammatory diseases and as such, serve as protein markers of oxidative stress [16,[38], [39], [40]].

In the last two decades, a body of evidence emerged that AOPPs are important modulators of the innate immune response and contribute to the potentiation and progression of oxidative stress and inflammation through stimulation of immune cells, specifically macrophages [[49], [50], [51], [52]]. HOCl-modified HSA (HSA_HOCl_) and other plasma proteins were found to increase ROS generation by monocytes and neutrophils at sites of inflammation [16,[39], [40], [41]] and recently we found that reversible N-chlorination is the main chemical modification that converts these proteins into potent activators of immune cells [16]. Furthermore, we observed that N-chlorinated HSA also acts as a pro-survival molecule that promotes neutrophil survival by binding to and reducing the uptake of highly immunogenic foreign antigens and thus, has the potential to delay clearance by macrophages [16]. The effects of HSA_HOCl_ on monocyte activity [39,41] and its ability to neutralize antigens prompted us to speculate that AOPPs could also influence the antigen processing and presenting capacity of macrophages at sites of infection.

Here, we study the effects of HSA_HOCl_ on the internalization and intracellular degradation of various antigens, as well as on the expression of MHC class II proteins by pro-inflammatory M1 macrophages. We show that endocytosis of the model antigen ovalbumin (OVA) decreased in the presence of increasing amounts of N-chlorinated HSA, with patho-physiologically relevant amounts of HSA_HOCl_ fully inhibiting receptor-mediated antigen uptake by these immune cells. Moreover, presence of N-chlorinated HSA drastically reduced the total expression of MHC II complexes by the macrophages and their ability to directly present antigenic peptide fragments, resulting in the reduced activation and proliferation of antigen-specific CD4^+^-T cells. The acquisition of pathogens and pathogen-derived antigens by macrophages at sites of infection, however, is an essential step in innate immunity to initiate a specific adaptive response against a foreign invader. Hence, we propose that the reduced antigen presentation via MHC class II proteins by macrophages, as observed in the presence of HSA_HOCl_ at concentrations feasible at sites of acute inflammation, shifts antigen presentation to sites removed from the initial inflammation event. Excessive retardation or complete inhibition of antigen uptake and presentation, however, could prolong disease by slowing down the immune system's response and thereby contribute to the development and progression of chronic infection and inflammation.

## 2 Material and Methods

### 2.1 Preparation of protein stock solutions

Lyophilized albumin from human serum (HSA, Product # A9511) and from chicken egg white (OVA, Product # A5503) was purchased from Sigma-Aldrich, St. Louis, USA, and used without further purification. HSA and OVA stock solutions were freshly prepared by dissolving varying amounts of protein in 1xPBS buffer, pH 7.4 (Gibco Life Sciences). Concentration of the HSA and OVA stock solutions was determined by measuring the absorbance at 280 nm (A_280nm_) using a JASCO V-650 UV/VIS spectrophotometer. The molar extinction coefficient used for HSA was *ε*_280_ = 35,700 M^−1^ cm^−1^ [42] and for OVA *ε*_280_ = 30,590 M^−1^ cm^−1^ [43].

### 2.2 Methylation of proteins

Proteins were methylated as previously described [44] with some modifications. Proteins were dissolved in 1 mL 1xPBS, pH 7.4 to a concentration of 10 mg ^**.**^ mL^−1^ and the solution cooled to 4 °C. 20 μL of 60 mg ^**.**^ mL^−1^ dimethylamine borane complex and 40 μL 1 M formaldehyde were then added. After 2 h of incubation at 4 °C, this step was repeated. 2 h later a final aliquot of 10 μL dimethylamine borane complex solution was added and the reaction mixture was incubated at 4 °C overnight. The next morning 125 μL of 1 M Tris pH 7.5 were added to stop the reaction. Other than the dimethylamine borane complex, no reductants, such as DTT or TCEP were added to the reaction mixture in order to preserve the structural disulfide bonds in HSA. The reacting agents were then separated from the now methylated proteins by size-exclusion chromatography using Nap™-5 columns (GE Healthcare Life Sciences, Amersham, UK) according to the manufacturer's instructions.

### 2.3 Treatment of proteins with HOCl and reduction

Proteins were exposed to HOCl as described previously [16]. Briefly, a fresh working solution of NaOCl was prepared immediately before each chlorination reaction by diluting a ~0.64 M NaOCl stock solution (Sigma-Aldrich, St. Louis, USA) with 1xPBS pH 7.4 to the desired concentration. The concentration of the NaOCl working solution was determined by measuring the absorbance at 292 nm using a JASCO V-650 UV/VIS spectrophotometer and the extinction coefficient *ε*_292_ = 350 M^−1^ cm^−1^.

1 mM HSA or 1 mM OVA was then treated with a 50-fold molar excess of NaOCl for 10 min at 30 °C and then purified with gel filtration using Nap™-5 columns according to the manufacturer's instructions. The concentration of HOCl-treated HSA (HSA_HOCl_) and HOCl-treated OVA (OVA_HOCl_) was determined as described above.

To reduce HOCl-treated proteins, sodium ascorbate, dithiothreitol (DTT) or methionine was dissolved in 1xPBS pH 7.4 to a concentration of 1 M (sodium ascorbate, DTT) or 140 mM (methionine) and the proteins were incubated with a 50-fold molar excess of these reductants for 45 min at 37 °C. After removal of excess reductant (see above), protein concentrations were again re-determined.

### 2.4 Quantification of chloramines using 3,3′,5,5′-tetramethylbenzidine

Quantification of chloramines on variously treated HSA samples was performed using the method of Dypbukt et al. (2005) [45] as described previously without any modifications [16]. Chloramine content was determined at different time points upon incubation of HSA at 30 °C in 1x PBS buffer solution, pH 7.4 and serum-free RPMI 1640 medium in the absence or presence of 1.9 × 10^5^ cells · mL^−1^ THP-1 cells, respectively. Cells were removed from the HSA solution by filtration prior to measurement. HSA_HOCl_ was used at a final concentration of 1 μM. HSA_UT_ and HSA_HOCl_ after reduction with sodium ascorbate, DTT or methionine were used at a final concentration of 10 μM. For quantification, a standard curve generated with known quantities of taurine monochloramine was used. Absolute number of monochloramines per molecule HSA was calculated by dividing the measured monochloramine concentration by the respective protein concentration.

### 2.5 Fluorescent labeling of proteins

For fluorescent labeling of native HSA (HSA_UT_) and HSA_HOCl_ or OVA_HOCl_ (see above), the fluorescent dyes CF™ 405S succinimidyl ester and CF™ 405S aminooxy or CF™ 488A aminooxy (Sigma-Aldrich, St. Louis, USA) were used, respectively. Succinimidyl ester groups label free amino groups of HSA_UT_, while aminooxy groups readily react with HOCl-introduced carbonyl groups in HSA_HOCl_ and OVA_HOCl_ to form a stable oxime linkage. Total carbonyl group content in HSA and OVA after HOCl treatment was quantified prior to labeling using a 2,4-dinitrophenylhydrazine (DNPH; Sigma-Aldrich, St. Louis, USA) assay [46] based on the derivatization of protein carbonyl groups (PCO) with DNPH followed by spectrophotometric measurements. The assay was performed following the step-by-step protocol provided by Colombo et al. [47] without any modifications.

Labeling of HSA_UT_, HSA_HOCl_ and OVA_HOCl_ was carried out according to the manufacturer's instructions with slight modifications. To increase labeling efficiency of HSA_HOCl_ and OVA_HOCl_, the labeling reaction was accelerated by adding 9 μL ^**.**^ mL^−1^ 10.6 M aniline solution (Sigma-Aldrich, St. Louis, USA) as catalyst [48] and carried out at 4 °C overnight under continuous shaking. Excess dye and catalyst were removed by size-exclusion chromatography using PD-10 desalting columns containing Sephadex G-25 resin according to the manufacturer's instructions (GE Healthcare Life Sciences, Amersham, UK). Concentration of the conjugate and degree of labeling (DOL) were calculated for the labeled proteins using the respective formula provided by the dye's manufacturer. For better comparison, the different DOL of HSA_UT_ and HSA_HOCl_ were adjusted to the same value by adding the respective unlabeled protein at the same molar concentration as the corresponding conjugate.

### 2.6 THP-1 cell culture and differentiation

THP-1 cells, a human acute leukemia monocytic cell line (certified mycoplasma negative, obtained from DSMZ, German collection of microorganisms and cell culture) [49], were cultured in RPMI 1640 medium supplemented with 10% heat-inactivated FCS and 1% GlutaMAX (Life Technologies, Carlsbad, CA) at 37 °C in a humidified 5% (v/v) CO_2_ atmosphere. THP-1 cells were kept at a minimum density of 2 × 10^5^ cells · mL^−1^ and were passaged every 3–4 days with a complete medium replacement after having reached a density of 8 × 10^5^ to 1 × 10^6^ cells · mL^−1^. For the differentiation into resting macrophages (M0), THP-1 cells were seeded in clear, flat-bottom 24-well or 96-well plates (Sarstedt, Nümbrecht, Germany) at 3.8 × 10^5^ cells · mL^−1^ (380,000 cells per well) and 2.5 × 10^5^ cells · mL^−1^ (50,000 cells per well), respectively. Differentiation was induced by adding phorbol-12-myristate 13-acetate (PMA; Sigma-Aldrich, St. Louis, USA) to a final concentration of 81 nM. After 72 h of incubation, the PMA supplemented medium was removed and the now adherent cells were rinsed three times with 1xPBS, before fresh PMA-free medium was added to the wells for 24 h before further treatment (M0 macrophages) [[50], [51], [52]]. Resting (M0) macrophages were then polarized toward the classically activated M1 phenotype by incubation for 24 h with 20 ng mL^−1^ (400 U · mL^−1^) IFN-γ (ImmunoTools, Friesoythe, Germany), followed by incubation for 24 more hours with 20 ng · mL^−1^ IFN-γ together with 10 ng · mL^−1^ LPS (Sigma-Aldrich, St. Louis, USA) [52]. For appropriate experiments, 10 ng · mL^−1^ TNF-α (Sigma-Aldrich, St. Louis, USA) were added to the cells instead of LPS. TNF-α was found to upregulate IFN-γ receptors on monocytes and macrophages and thus, increase the capacity of IFN-γ to activate macrophages and induce expression of MHC class II molecules (i.e. HLA-DR) [53].

The viability of the differentiated cells was evaluated using trypan blue dye and was typically >95% after 24 h of incubation with IFN-γ and then >80% after another day of incubation with IFN-γ and LPS or TNF-α. The obtained M1-type macrophages were confirmed to have characteristic macrophage cell morphology by light microscopy and significantly increased expression of the cell surface marker CD14, as evaluated by flow cytometry using anti-CD14 monoclonal antibodies [54] (Supplementary Fig. S1).

### 2.7 Cell viability assay

For the analysis of cell viability in the presence of HSA, various amounts of native HSA (HSA_UT_), HSA_HOCl_, or buffer were added to THP-1 monocytes in serum-free RPMI 1640 medium. The maximum HSA concentration tested was 300 μM and corresponded to the highest amount of HSA that has been reported within interstitial fluids [55,56]. Upon incubation for 2, 4 or 6 h, the cells were washed once with cold 1xPBS, followed by one washing step with 1x Annexin V binding buffer. The variously treated cell suspensions were then stained with Annexin V-FITC and propidium iodide (PI) using the Dead Cell Apoptosis Kit (Invitrogen, Thermo Fisher Scientific, Waltham, Massachusetts, USA) according to the manufacturer's instructions and subsequently subjected to flow cytometry. Samples were analyzed using a BD FACSCanto II flow cytometer (Becton, Dickinson and Company). Fluorescence emitted by Annexin V-FITC and PI was measured through a 530/30-nm and 585/42-nm bandpass filter, respectively, upon excitation with an argon ion laser operating at 488 nm. Single-stained compensation controls were used to calculate the compensation matrix. 30,000 events were acquired and recorded per sample. Data were analyzed using FlowJo (version 10) software.

### 2.8 T cell proliferation assay

To directly analyze the presentation of a particular antigen to antigen-specific CD4^+^-T cells via MHC class II proteins, a cell proliferation assay using Tetanus toxoid (TT)- specific CD4^+^-T cells was performed. A single-use vial of human TT-specific CD4^+^- T cells (Lot no. 4062OC18; female donor: ID213) and TT (Lot no. 2002AU18) were purchased from Astarte Biologics, Bothell, WA, US. 50'000 THP-1-derived M1 macrophages (APCs) were incubated with 10 μg mL^−1^ TT antigen in serum-free RPMI medium containing 20 ng · mL^−1^ IFN-γ and 10 ng · mL^−1^ TNF-α for 5 h at 37 °C. Incubation with TT occurred in the absence or presence of 50 μM HSA_UT_ and HSA_HOCl_, respectively. The different media were then replaced with fresh serum-free RPMI medium supplemented with 50 μg mL^−1^ mitomycin C (Sigma-Aldrich, St. Louis, USA) to stop proliferation of the APCs. After incubation for 30 min at 37 °C, the APCs were washed extensively with 1xPBS, before 20'000 TT-specific CD4^+^- T cells in RPMI containing 10% FCS were added. As a positive control, antigen receptor stimulation of the T cells leading to T cell activation and expansion was achieved in the absence of APCs using an antigen mimicking, immobilized anti-CD3 antibody in combination with soluble anti-CD28 antibodies [57]. For this, the respective wells in a transparent, flat-bottom 96-well plate (Sarstedt, Nümbrecht, Germany) were first coated with 1.5 μg mL^−1^ anti-CD3 (clone OKT3; Miltenyi Biotec, Bergisch Gladbach, Germany) over night at 4 °C, before 20'000 T cells in RPMI medium containing 10% FCS and 1 μg mL^−1^ anti-CD28 antibody (clone 15E8; Miltenyi Biotec, Bergisch Gladbach, Germany) were added to the wells. The 96-well plate was sealed with a Breathe-Easy membrane (Sigma-Aldrich, St. Louis, USA) and T cell proliferation was measured 3, 5 and 7 days after stimulation using CellTiter-Glo Luminescent cell viability assay (Promega, Madison, WI, US). 50 μL cell suspension from each well were transferred to a non-transparent, white, flat-bottom 96-well plate (Pierce™, Thermo Fisher Scientific, Waltham, MA, USA) and mixed with 50 μL CellTiter-Glo reagent according to the manufacturer's instructions. After 10 min of incubation at room temperature, chemiluminescence of the various cell samples was measured in triplicates using the Synergy H1 multi-detection microplate reader (Biotek, Bad Friedrichshall, Germany).

### 2.9 Uptake of fluorescently labeled exogenous antigens

380,000 THP-1-derived M1 macrophages were incubated with the indicated amounts of a fluorescently-labeled antigen in the presence or absence of various amounts of native and variously treated HSA in serum-free RPMI supplemented with 20 ng · mL^−1^ IFN-γ and 10 ng · mL^−1^ TNF-α at 37 °C for the indicated time periods. For some experiments, cells were pre-treated with 10 μg mL^−1^ CD36-specific blocking antibody (clone JC63.1; Abcam; ab23680) for 30 min at 37 °C prior to the addition of the antigen and HSA. Negative controls were treated in the same way but kept on ice to inhibit receptor-mediated endocytosis of the antigen [58]. Antigens used were: OVA conjugated to Alexa Fluor 488 (OVA-488) purchased from Invitrogen (Thermo Fisher Scientific, Waltham, MA, USA), HOCl-treated OVA labeled with CF™ 488A aminooxy (OVA_HOCl_-488; see above) and mycobacterial Ag85B, heterologously expressed in *Escherichia coli* BL21 (DE3), purified and labeled with CF™ 488A succinimidyl ester (Sigma-Aldrich, St. Louis, USA) (Ag85B-488), as described previously [16]. After the respective incubation time, cells were washed three times with 1xDBPS and stained with PI using the Dead Cell Apoptosis Kit (see above). After three washing steps, cells were fixed with 4% paraformaldehyde (PFA) for 10 min on ice, washed again, detached from the plate using Mini Cell Scrapers and then immediately analyzed by flow cytometry using a BD FACSCanto II flow cytometer. Fluorescence emitted by OVA-488, OVA_HOCl_-488 or Ag85B-488 and PI was measured through a 530/30-nm and 585/42-nm bandpass filter, respectively, upon excitation with the 488 nm argon laser line. Single-stained compensation controls were used to calculate the compensation matrix. Cells that were PI positive were excluded from the analysis. The median fluorescence intensity of the ice control cells was subtracted from that of cells incubated at 37 °C.

### 2.10 Uptake of fluorescent human serum albumin

To analyze the uptake of fluorescently labeled HSA_UT_ (HSA_UT_-405) and HSA_HOCl_ (HSA_HOCl_-405) by macrophages, 380,000 THP-1-derived M1 macrophages were incubated with 0.75 μM HSA_UT_-405 or HSA_HOCl_-405 in serum-free RPMI medium for 3 h at 37 °C. The cells were then washed three times with 1xDPBS, followed by fixation with 4% PFA for 10 min on ice. After fixation, cells were again washed three times with 1xDPBS, harvested from the well plate by scraping and subsequently subjected to flow cytometry. Fluorescence emitted by HSA_UT_-405 and HSA_HOCl_-405 was measured through a 450/50-nm bandpass filter upon excitation with the 405 nm violet laser line.

### 2.11 Intracellular antigen processing

Intracellular degradation (processing) of antigens by THP-1-derived M1 macrophages was examined using BODIPY-conjugated DQ-OVA (Invitrogen, Thermo Fisher Scientific, Waltham, MA, USA), a self-quenched conjugate of OVA that exhibits green fluorescence only upon denaturation and proteolytic cleavage releasing the dye from OVA. 380'000 THP-1-derived M1 macrophages were incubated with 50 μg mL^−1^ DQ-OVA in 1xPBS for 15 min at 37 °C and then washed three times with ice-cold 1xPBS to remove excess protein. Afterwards, native or variously treated HSA or the respective volume of 1xPBS (buffer control) in serum-free RPMI containing 20 ng · mL^−1^ IFN-γ and 10 ng · mL^−1^ TNF-α was added to the cells. The final concentration of HSA in all samples was 50 μM. Negative controls were treated in the same way but kept on ice to allow surface binding of DQ-OVA but no internalization. To measure antigen degradation over time, the assay was carried out at 30 min, 1 h and 2 h (in triplicates per treatment and time-point). After each time-point, cells were washed three times with 1xPBS, fixed with 4% PFA for 10 min on ice, washed again, detached from the plate by scraping, and then subjected to flow cytometry. Fluorescence of the variously treated cells was measured through a 530/30-nm bandpass filter upon excitation with the 488 nm argon laser line. The median fluorescence intensity of the ice control cells was subtracted from that of cells incubated at 37 °C.

### 2.12 Visualization of antigen processing in live cells

To visualize intracellular processing of antigens by macrophages, 100'000 THP-1-derived M1 macrophages in a chambered 8-well, tissue culture treated polymer coverslip (μ-Slide 8 Well, ibiTreat; ibidi, Gräfelfing, Germany) were first stained with CellTracker™ Red CMTPX Dye (Invitrogen, Thermo Fisher Scientific, Waltham, MA, USA) for 30 min at 37 °C. After staining, the cells were pre-incubated with BODIPY-conjugated DQ-OVA for 15 min at 37 °C, before HSA_UT_, HSA_HOCl_, or buffer was added to the cells as described above. Processing of DQ-OVA in the presence or absence of HSA was subsequently monitored in real-time over a period of 1.5 h at 37 °C by live-cell fluorescence microscopy.

Fluorescence images were acquired every few minutes with an LSM 880 ELYRA PS.1 microscope (Carl Zeiss Microscopy GmbH, Jena, Germany). Images were acquired in different channels according to the fluorophore: to detect BODIPY-conjugated DQ-OVA (Ex_488nm,_ Em_490–553nm_) and to detect CellTracker™ Red CMTPX Dye (Ex_561nm_, Em_566–698nm_). Individual single channel images were exported using ZEN 2.1 (Zeiss, DE).

### 2.13 Flow cytometry-based analysis of MHC class II expression

To test the effect of native or modified HSA on IFN-γ-induced expression of MHC class II molecules, varying amounts of the proteins were added to THP 1-derived M0 macrophages in serum-free RPMI medium immediately before M1 polarization was induced with 20 ng · mL^−1^ IFN-γ. After up to 6 h of incubation with IFN-γ in the presence of the different proteins, the cells were then washed three times with 1xDPBS and incubated for 30 min on ice with human Fc-γ receptor block antibodies (Invitrogen, Thermo Fisher Scientific, Waltham, MA, USA) prior to staining. 10 μg mL^−1^ anti-HLA-DR (clone L243; Santa Cruz Biotechnology, Heidelberg, Germany) labeled with Alexa Fluor-647 or the corresponding isotype control in 1xPBS containing 5% bovine serum albumin (BSA) was added to the cells and incubated for 1 h on ice. The cells were then washed three times with 1xDPBS, before the M1 macrophages were harvested from the plate and subsequently subjected to flow cytometry. Fluorescence of the variously treated cells was measured through a 660/20-nm bandpass filter upon excitation with the 633 nm red laser line.

### 2.14 Analysis of total protein amounts by western blotting

To analyze the effect of HSA_HOCl_ on the total protein expression in whole cells, M0 macrophages were polarized into M1-type macrophages with 20 ng · mL^−1^ IFN-γ in the presence of various amounts of native or modified HSA as indicated and incubated for up to 48 h. The cells were then harvested from the plate and washed three times with 1xPBS. Cells were lysed with Pierce™ RIPA buffer (Thermo Fisher Scientific, Waltham, MA, USA) containing cOmplete™ Mini EDTA-free protease inhibitor cocktail (Sigma-Aldrich, St. Louis, USA) and disrupted through sonication (amplitude: 80%, Cycle: 0.5, 4 × 1 min) using a Vial Tweeter (Hielscher, Teltow, Germany). The resulting crude extract was centrifuged (10,000×*g*, 45 min, 4 °C) to remove cell debris. Protein concentrations in the supernatants were determined using the Pierce™ Micro BCA protein assay kit (Thermo Fisher Scientific, Waltham, MA, USA) according to the manufacturer's instructions. 75–150 μg of protein were loaded and separated on 4–12% Bis-Tris Gels (NuPAGE™, Invitrogen, Carlsbad, CA) under reducing conditions (80 V). For some experiments the same amount of protein extract (i.e. equivalent number of extracted cells) was loaded. For Western blot analysis, proteins were transferred onto nitrocellulose membranes using the iBlot™ 2 Dry Blotting System (Invitrogen). Membranes were blocked with 5% non-fat dry milk in 1x Tris-buffered saline (TBS) for 1 h, before they were probed with antibodies against MHC class II (mouse monoclonal: clone TAL 1B5; Santa Cruz Biotechnology, Heidelberg, Germany; 1:200), ubiquitin (mouse monoclonal: clone P4D1; Santa Cruz Biotechnology, Heidelberg, Germany; 1:200), CIITA (mouse monoclonal: clone 7-1H; Santa Cruz Biotechnology, Heidelberg, Germany; 1:200), or β-actin (rabbit monoclonal: clone SP124, ab115777, 1:200; Abcam) in TBS containing 2% non-fat dry milk at 4 °C overnight. Membranes were then washed and incubated with fluorescent secondary anti-mouse (from donkey, 1:5000, IRDye 800CW, LICOR, NE) or anti-rabbit antibodies (from goat, 1:5000, IRDye 680RD, LICOR, NE) for 1 h in TBS containing 2% non-fat dry milk. Blots were imaged with an infrared imaging system (Odyssey Classic, LICOR, NE) and analyzed using ImageJ. Relative densities of the β-actin, MHC class II, CIITA and ubiquitin bands were calculated separately. Relative densities of MHC class II, CIITA and ubiquitin bands were then normalized to β-actin by dividing the relative density of the respective protein band by the relative density of the corresponding β-actin band of the same sample.

### 2.15 Visualization of MHC class II cell surface expression

Evaluation and visualization of MHC class II cell surface expression by THP-1-derived M1 macrophages was also carried out at single-cell level using high-resolution, confocal fluorescence microscopy. 100'000 THP-1-derived M0 macrophages in a chambered 8-well, tissue culture treated polymer μ-Slide coverslip (ibiTreat) were polarized into M1 macrophages in the absence or presence of 300 μM HSA_UT_ or HSA_HOCl_ in serum-free RPMI medium by adding 20 ng · mL^−1^ IFN-γ. After 6 h of incubation, cells were washed three times with 1xDPBS. Cytoplasm of the cells was stained with CellTracker™ Green CMFDA Dye (Invitrogen, Thermo Fisher Scientific, Waltham, MA, USA) for 30 min at 37 °C. After staining, the cells were treated in the same way as described above to detect MHC class II molecules at the cell surface. After fixation and three washing steps with 1xDPBS, cell nuclei were stained with DAPI and the cells were subsequently subjected to fluorescence microscopy. Fluorescence images of the variously treated cells were acquired with an LSM 880 ELYRA PS.1 microscope in different channels according to the fluorophore: to detect DAPI (Ex_405nm_/Em_410–483nm_); to detect CellTracker™ Green CMFDA Dye (Ex_488nm_/Em_490–624nm_) and to detect Alexa Fluor 647 (Ex_633nm_/Em_638–755nm_). Images were exported using ZEN 2.1.

### 2.16 Protein stability assay

To analyze the stability of MHC class II proteins in the presence of HSA_UT_ and HSA_HOCl_, THP-1-derived M1 macrophages were pre-incubated with 15 μg mL^−1^ cycloheximide (CHX; Sigma-Aldrich, St. Louis, USA), a protein synthesis inhibitor, or vehicle (DMSO) for 1 h, before 50 μM HSA_UT_ or HSA_HOCl_ in RPMI medium supplemented with 10% FCS and 20 ng · mL^−1^ IFN-γ were added to the cells for the indicated time periods. The cells were then detached from the plates and washed three times with 1xPBS. MHC class II protein levels were then analyzed by Western blotting as described above and normalized to the level of β-actin.

### 2.17 Statistical analysis

Experiments were typically performed in biologically independent triplicates. Due to the biological variances and the gain settings used during our flow cytometry experiments, the data was normalized as % fraction of the fluorescence of the corresponding control, where appropriate. The quantification of western blot data was performed in a similar manner. Due to this, the standard deviation of the control was not calculated in these experiments (Fig. 3 d, f; 4 d, f, I, k; 6 d, f, i; 7 b, c). Data were otherwise expressed as mean ± standard deviation. Statistical significance of differences between the means of two unpaired samples was determined using the two-sided Welch's two independent sample *t*-test for unequal variances [59,60]. Two-tailed Welch's *t*-test was performed using the *t*-test function “Two-Sample Unequal Variance” in Microsoft Excel. For multiple comparisons of the group means in the same data set, a one-way Analysis of Variance (ANOVA) test was performed using GraphPad Prism, version 9. In case of a significant P value, a Tukey or Dunnett post-hoc test was performed after the respective one-way ANOVA test. Differences between the means were considered as statistically significant at P values < 0.05.

## 3 Results

### 3.1 HOCl-treated human serum albumin leads to a dose- and time-dependent decrease in monocyte viability

Macrophages operate in environments rich in plasma proteins, at which neutrophils might have produced substantial amounts of HOCl. Thus, we first examined whether human serum albumin, exposed to HOCl (HSA_HOCl_) generally affects monocyte viability in the setting of chronic inflammation.

Under chronic inflammatory conditions, tissues are thought to be exposed to up to 25–50 mM of HOCl [61]. To mimic a state of chronic inflammation, HSA was thus treated with a 50-fold molar excess of HOCl corresponding to the maximum reported concentration of 50 mM, to generate AOPP-HSA (HSA_HOCl_). Inspection of HSA_HOCl_ on a reducing and non-reducing SDS gel did not reveal appreciable fragmentation (Supplementary Fig. S2) and since excess HOCl was removed through size exclusion chromatography, we could exclude the presence of insoluble aggregates in our HSA_HOCl_ preparation.

In our studies, we used the human monocyte-like cell line THP-1, which resembles primary blood monocytes in morphology, differentiation properties, and selective functions [49,62]. We incubated THP-1 monocytes in the presence of various concentrations of HSA_HOCl_ ranging from 50 μM to 300 μM, which correspond to the amounts of native HSA that can be typically found in interstitial fluids [55,56].

**Fig. 1:**
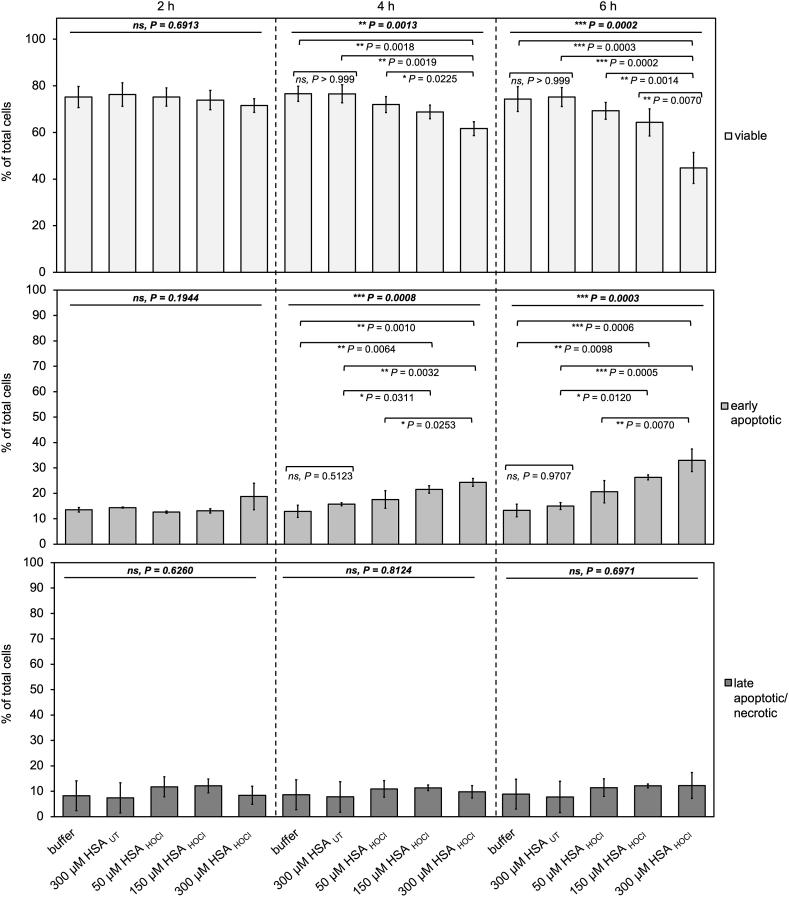
**Exposure of THP-1 monocytes to HSA**_**HOCl**_**leads to a dose- and time-dependent decrease of the ratio of viable to early apoptotic cells**. THP-1 monocytes were incubated with 300 μM HSA_UT_ or various concentrations of HSA_HOCl_ for 2, 4, or 6 h, before they were stained with Annexin V-FITC and propidium iodide (PI) to identify viable, early apoptotic and late apoptotic/necrotic cells. Cells treated only with buffer served as control. Data are shown as the mean ± standard deviation of three independent experiments. One-way ANOVA test was performed to determine the significance of the differences between the group means in one data set. The resulting P value is indicated in bold and italic above the line at the top of each data set. If P < 0.05, a Tukey post-hoc test was performed. The resulting P values for the pair-wise comparison of the outermost 2 bars within the individual brackets are shown. P values < 0.05 indicate statistical significance; *P < 0.05; **P < 0.01; ***P < 0.001. ns = not significant.

After incubation for two, four, or 6 h, viability of the variously treated cells was evaluated by flow cytometry using Annexin V-FITC/propidium iodide (PI) staining which allows discrimination of viable, early apoptotic, and late apoptotic/necrotic cells through differences in plasma membrane integrity and permeability [63] (Fig. 1). A representative set of data plots, generated from analysis of ungated data, is shown in Supplementary Fig. S3 a. Notably, THP-1 monocytes exhibited a reduced viability of 75–80%, an average apoptosis rate of 18% and a necrosis rate of up to 8% already at the beginning of our experiments (time point “0”; see Supplementary Fig. S3 b). A percentage of apoptotic/necrotic cells of about 20% under steady-state conditions has also been reported by others [64,65].

Exposure to HSA_HOCl_ led to a dose- and time-dependent decrease of the ratio of viable to early apoptotic cells (Fig. 1). While HSA_UT_, added at the maximum reported interstitial concentration of 300 μM had no effect on monocyte viability throughout the entire incubation period, concentrations of HSA_HOCl_ exceeding 150 μM progressively decreased the number of viable cells, while increasing the amount of early apoptotic cells. Notably, presence of HSA_HOCl_, even at the highest concentration, did not increase necrotic cell death, suggesting that HSA_HOCl_, when present in sufficient amount, affects lifespan of newly recruited monocytes through inducing apoptosis rather than promoting direct cell death e.g. due to the oxidizing properties of HSA_HOCl_ itself. HSA_HOCl_ may therefore contribute to the regulation of the monocyte pool in a ROS-enriched inflammatory environment to reduce the overall increased ROS levels and contain inflammation-induced tissue damage.

### 3.2 HOCl-modified serum albumin decreases the macrophages’ ability to present antigens to antigen-specific CD4^+^ T cells

Upon migration into infected tissues, monocytes then differentiate into pro-inflammatory M1 macrophages, capable of presenting antigens to T cells via MHC class II molecules [66].

**Fig. 2:**
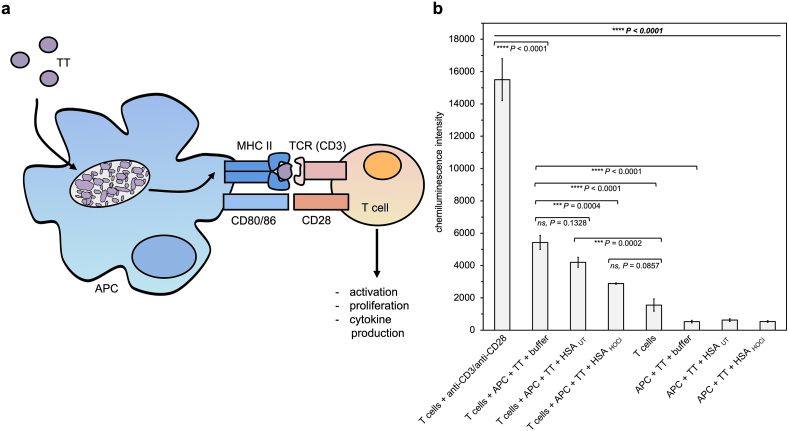
**HOCl-modified human serum albumin reduces MHC class II-dependent presentation of antigens to antigen-specific T cells by THP-1-derived M1 macrophages.** (**a**) Schematic overview of the activation of Tetanus toxoid (TT)-specific CD4^+^- T helper cells upon recognition of a TT fragment bound to MHC class II molecules of THP-1-derived M1 macrophages (APC). Upon internalization and degradation of TT by APCs, peptide fragments are loaded onto MHC class II proteins and presented at the cell surface. Peptide-specific T cells recognize a particular TT fragment via the T cell receptor (TCR/CD3). T cell activation requires a second, co-stimulatory signal provided by the interaction of T cell membrane protein CD28 with CD80 or CD86 of the APC. Successful activation triggers proliferation of the T cells and cytokine production. (**b**) Proliferation of TT-specific CD4^+^- T helper cells 5 days after stimulation. T cells were left untreated or either stimulated by anti-CD3/CD28 or by mitomycin C-treated APCs, which had been pre-incubated with TT antigen in the absence (buffer) or presence of HSA_UT_ or HSA_HOCl_. T cell proliferation was evaluated by performing a CellTiter-Glo Luminescent Cell viability assay. Chemiluminescence of the various cell samples was measured in triplicates and means and standard deviations are shown. One-way ANOVA test followed by a Tukey post-hoc test was performed to determine the significance of the differences between the group means in one data set. The resulting P value of the one-way ANOVA test is indicated in bold and italic above the line at the top of the data set. The P values of the post-hoc test for the pair-wise comparison of the outermost 2 bars within the individual brackets are shown. P values < 0.05 indicate statistical significance; ***P < 0.001; ****P < 0.0001. ns = not significant. APC = antigen presenting cell; TT = Tetanus toxoid; TCR = T cell receptor.

The direct outcome of successful antigen presentation by macrophages is the activation and proliferation of antigen-specific CD4^+^ T cells. To test, whether HSA_HOCl_ has any effect on the antigen presenting capacity of M1 macrophages, we first developed an *in vitro* cell-based antigen presentation assay to study T cell proliferation upon stimulation with antigen-bearing antigen-presenting cells (Fig. 2 a).

**Fig. 3:**
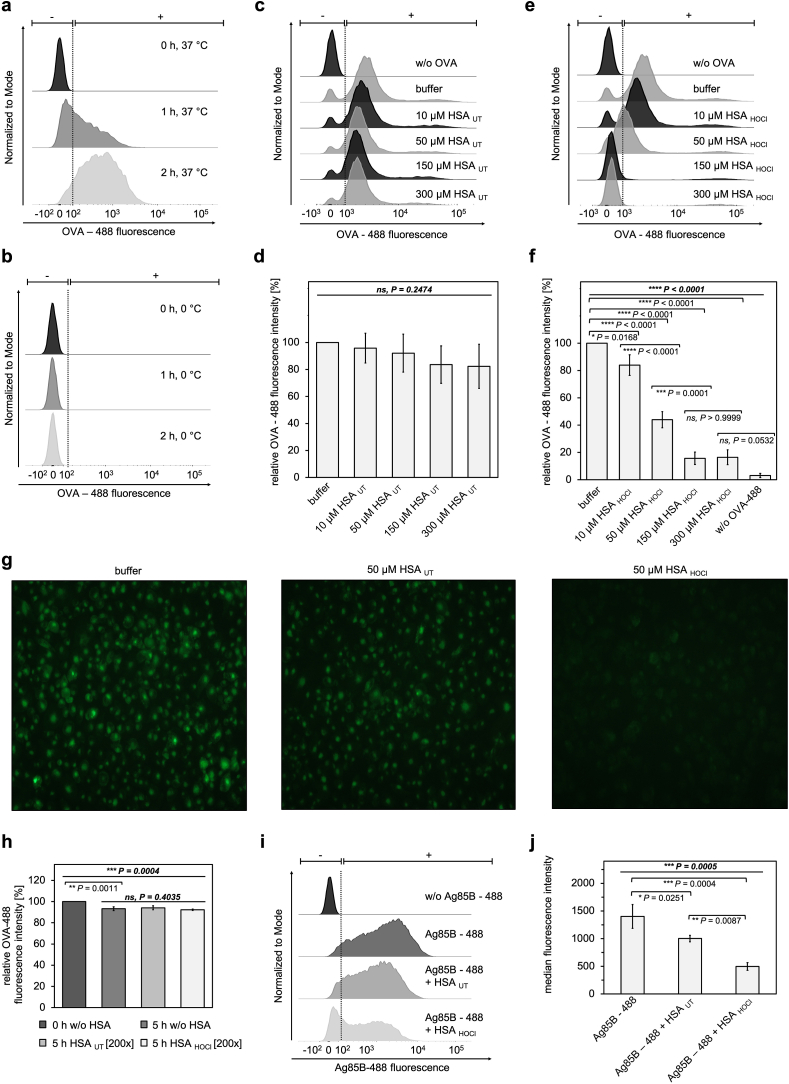
**HOCl-modified human serum albumin decreases uptake of antigens by THP-1-derived M1 macrophages in dose-dependent manner.** THP-1-derived M1 macrophages were incubated with 50 μg mL^−1^ fluorescently-labeled ovalbumin (OVA-488) in the absence of HSA at **(a)** 37 °C or **(b)** 0 °C, or together with various amounts of (**c**, **d**) HSA_UT_ or (**e**, **f**) HSA_HOCl_. After incubation for up to 2 h, uptake of fluorescent OVA-488 by the variously treated cells was assessed by flow cytometry. **(g)** Visualization of OVA-488 uptake by fluorescence microscopy after 2-h incubation in the presence of HSA_UT_, HSA_HOCl_ or buffer. Exposure time: 100 ms. **(h)** OVA-488 was incubated in the absence (w/o HSA) or in the presence of 200-fold molar excess of HSA_UT_ or HSA_HOCl_ for 5 h. Fluorescence intensity of OVA-488 prior to the addition of HSA was set to 100%. **(i, j)** THP-1-derived M1 macrophages were incubated with fluorescently labeled Ag85B (Ag85B-488; 3.1 μM) and HSA_UT_ or HSA_HOCl_, added in a 50-fold molar excess over Ag85B, for 1 h. Uptake of fluorescent Ag85B-488 by the variously treated cells was then assessed by flow cytometry. **(a, b, c, e, i)** Single-parameter histogram overlays of OVA-488 or Ag85B-488 fluorescence of the various samples are shown. **(d, f, h, j)** Results of three experiments are shown (means and standard deviations). Uptake of OVA-488, reflected by the median fluorescence intensity, in the absence of HSA (buffer) was set to 100%. One-way ANOVA test was performed to determine the significance of the differences between the group means in one data set. The resulting P value is indicated in bold and italic above the line at the top of each data set. If P < 0.05, a Tukey post-hoc test was performed. The resulting P values for the pair-wise comparison of the outermost 2 bars within the individual brackets are shown. P values < 0.05 indicate statistical significance; *P < 0.05; **P < 0.01; ***P < 0.001; ****P < 0.0001. ns = not significant.

In this assay, THP-1-derived M1 macrophages internalize the extrinsically applied antigen Tetanus toxoid (TT) by endocytosis, and after processing of the antigen, present fragments on their MHC II receptor to human primary Tetanus toxoid-specific CD4^+^ T cells. Upon recognition of the peptide-MHC II complex the T cell becomes activated and proliferates which can be analyzed by assessing the number of viable cells via a ATP-dependent luciferase reaction [67].

To test, whether HSA_HOCl_ affects the presentation process of TT to TT-specific CD4^+^ T cells by the M1 macrophages, cells were incubated with TT in the presence of HSA_UT_ or HSA_HOCl_ for 5 h, before they were used to stimulate the T cells. To exclude cytotoxic effects of HSA_HOCl_, as observed previously, HSA was added to a final concentration of 50 μM. As a positive control, antigen receptor stimulation of the T cells was achieved in the absence of an antigen-presenting cell using anti-CD3 and anti-CD28 antibodies to artificially mimic TCR and CD28 engagement, respectively [57]. After five days of co-culture with the variously treated M1 macrophages or anti-CD3/CD28, T cell proliferation was assessed as described above. The results of viable cell counts in these cultures are shown in Fig. 2 b. In line with expectations, stimulation with anti-CD3/CD28 antibodies induced a strong proliferative response resulting in clearly increased cell number compared to non-stimulated, resting T cells. Co-incubation with antigen-pulsed M1 macrophages likewise led to significant T cell expansion, albeit at a markedly lower rate compared to that induced by anti-CD/CD28. Presence of native HSA throughout the incubation period of the macrophages with TT, had no significant impact on the T cell response to the respective APC cultures. Pre-treatment of HSA with HOCl prior to the addition to the macrophages, however, clearly reduced the antigen presenting capacity of the APCs. T cell proliferation upon stimulation with APCs, which internalized and processed TT in the presence of HSA_HOCl_, was up to 50% lower compared with those T cells that were co-cultured with APCs pre-incubated with TT in the absence of HSA.

These results provide first hints, that HSA_HOCl_, present in low, non-cytotoxic doses, diminishes antigen-presenting capability of pro-inflammatory M1 macrophages and thus, probably also the capacity of the immune system to develop efficient adaptive responses to infection.

### 3.3 HOCl-modified serum albumin reduces antigen uptake by pro-inflammatory macrophages in dose-dependent manner

We previously found that binding of HSA_HOCl_ to exogenous antigens hindered their internalization by neutrophils. We, thus, suspected that HSA_HOCl_ could also inhibit antigen uptake by macrophages. To test this hypothesis, we added the fluorescently-labeled model antigen ovalbumin (OVA-488) to THP-1-derived M1 macrophages together with various amounts of HSA_UT_ or HSA_HOCl_ and incubated the cells for 2 h at 37 °C, before we analyzed OVA-488 uptake using flow cytometry. Negative controls were treated in the same way but kept on ice to inhibit endocytosis of the antigen [58]. As expected, no antigen uptake was observed upon incubation of the cells at 0 °C (Fig. 3 a, b). Internalization of OVA by the macrophages was not significantly affected by the presence of HSA_UT_, suggesting that native HSA rather does not interfere with antigen uptake (Fig. 3 c, d and g). Cells co-incubated with HSA_HOCl_, however, accumulated markedly less OVA-488 than did cells treated with OVA-488 alone (Fig. 3 e, f and g). The inhibitory effect of HSA_HOCl_ on OVA-488 uptake was dose-dependent, as the extent of OVA-488 internalization successively decreased with increasing amount of HSA_HOCl_ added. Strikingly, no significant antigen uptake could be observed when HSA_HOCl_ was present at a concentration of at least 150 μM, suggesting that physiologically relevant amounts of HSA_HOCl_ that can be typically found within inflamed tissues can fully inhibit the endocytosis of antigens. To confirm that the observed reduction in cell fluorescence is not due to a quenching effect of oxidized HSA, we incubated OVA-488 with a high molar excess of HSA_UT_ or HSA_HOCl_ (200:1) in the absence of cells and no significant reduction of the fluorescence signal could be observed (Fig. 3 h). Moreover, reduction in antigen uptake in the presence of HSA_HOCl_ was not restricted to one particular antigen, as similar results were obtained with fluorescently-labeled mycobacterial antigen Ag85B (Fig. 3 i, j). These findings strongly support the idea that inhibition of antigen uptake represents at least one possible mechanism by which HSA_HOCl_ reduces the ability of macrophages to present exogenous antigens.

### 3.4 HOCl-modified serum albumin likely interferes with scavenger receptor-mediated endocytosis of antigens

**Fig. 4:**
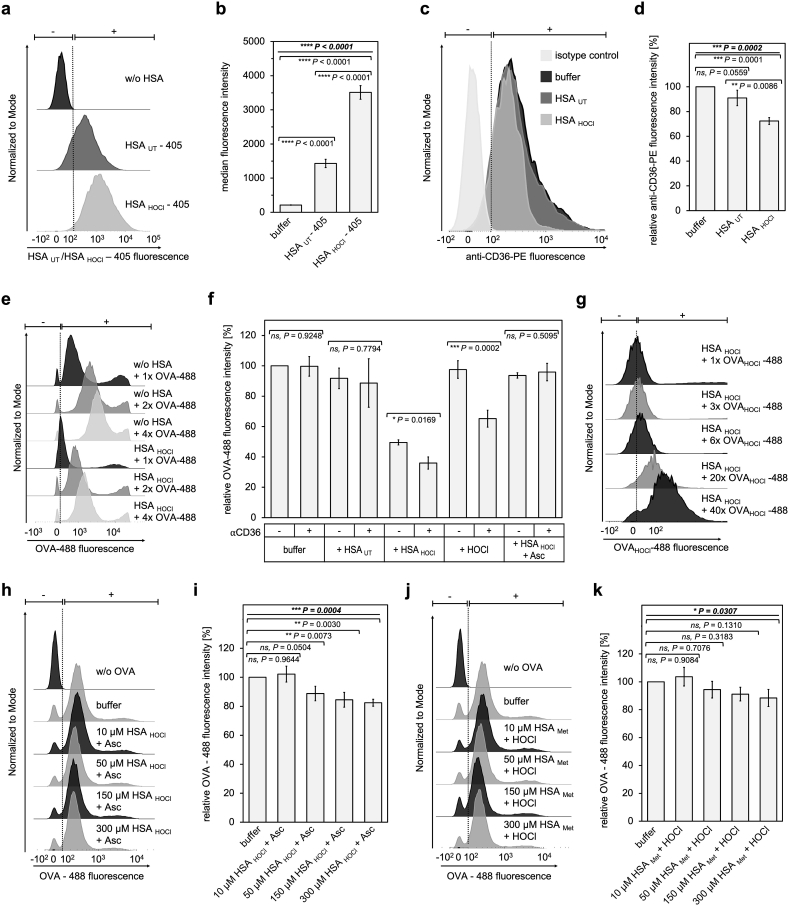
**Inhibition of antigen uptake by HOCl-modified HSA is likely due to an increased binding affinity to scavenger receptor CD36 conferred by reversible HOCl-induced N-chlorination. (a, b)** Fluorescently-labeled HSA_UT_ (HSA_UT_-405) or HSA_HOCl_ (HSA_HOCl_-405) was added to THP-1-derived M1 macrophages for 3 h. Different degrees of labeling of HSA_UT_-405 and HSA_HOCl_-405 were adjusted to the same value by adding unlabeled protein to the solution. **(c, d)** THP-1-derived M1 macrophages were pre-incubated with 50 μM HSA_UT_, HSA_HOCl_ or buffer for 15 min, before the cells were stained with a PE-conjugated anti-CD36 blocking antibody and analyzed by flow cytometry. **(e)** THP-1-derived M1 macrophages were incubated with 50 μg mL^−1^ (1x), 100 μg mL^−1^ (2x) or 200 μg mL^−1^ (4x) OVA-488 in the absence (w/o HSA) or presence of 50 μM HSA_HOCl_ for 2 h **(f)** THP-1-derived M1 macrophages were pre-incubated with anti-CD36 blocking antibody (+) or buffer (−), before 50 μg mL^−1^ OVA-488 together with 50 μM HSA_UT_, HSA_HOCl,_ HOCl, HSA_HOCl_ + 1 mM ascorbate (HSA_HOCl_ + Asc), or buffer were added to the cells for 2 h. **(g)** THP-1-derived M1 macrophages were incubated with 50 μg mL^−1^ (1x), 150 μg mL^−1^ (3x), 300 μg mL^−1^ (6x), 1 mg mL^−1^ (20x), or 2 mg mL^−1^ (40x) OVA_HOCl_-488 in the presence of 50 μM HSA_HOCl_ for 2 h. Differences in total fluorescence intensity of cells incubated with OVA-488 and OVA_HOCl_-488, respectively, are due to a markedly lower degree of labeling of OVA_HOCl_-488 compared to OVA-488. **(h**–**k)** Inhibitory effect of HSA_HOCl_ on the uptake of OVA-488 could be reversed by reduction with ascorbate (HSA_HOCl_ + Asc) and was largely abrogated by methylation prior to HOCl exposure (HSA_Met_ + HOCl). Uptake of the different proteins by the cells was analyzed by flow cytometry. **(a, c, e, g, h, j)** Single-parameter histogram overlays of HSA_UT_-405/HSA_HOCl_-405, OVA-488/OVA_HOCl_-488 or anti-CD36-PE fluorescence are shown. **(b, d, f, i, k)** Results of three experiments are shown (means and standard deviations). Uptake of OVA-488 in the absence of HSA (buffer) was set to 100%. **(f)** Welch's two independent sample *t*-test for unequal variances was performed to determine the significance of the effect of CD36 blocking (±) on OVA-488 uptake in the presence of the respective HSA preparation/HOCl. **(b, d, i, k)** One-way ANOVA test followed by a **(b, d)** Tukey or **(i, k)** Dunnett post-hoc test was performed to determine the significance of the differences between the group means in one data set. The resulting P value of the one-way ANOVA test is indicated in bold and italic above the line at the top of each data set. The P values of the post-hoc tests for the pair-wise comparison of the outermost 2 bars within the individual brackets are shown. P values < 0.05 indicate statistical significance; *P < 0.05; **P < 0.01; ***P < 0.001; ****P < 0.0001. ns = not significant.

Since receptor-dependent uptake of antigens has been reported to require 1000-fold less antigen than uptake via nonspecific pinocytosis to produce an equivalent T cell response, binding of antigens to cell surface receptors is a crucial initial step toward efficient antigen presentation [68]. To this end, macrophages express a wide variety of cell surface receptors to acquire antigens including mannose receptor (MC/CD206) and scavenger receptors (SR), such as the class B scavenger receptors type 1 (SR-B1) and type 2 (CD36) or scavenger receptor class A, type 1 (SR-A) [69,70]. Our observation that HSA_HOCl_ inhibits antigen uptake in a dose-dependent manner led us to speculate that HSA_HOCl_ may compete for the same receptor binding site that the antigen occupies and thus, is able to entirely prevent its internalization when present in sufficiently high amounts. To test, whether HSA_HOCl_ exhibits higher affinity to scavenger receptors and, thus, becomes internalized more efficiently than the native protein, we labeled HSA_UT_ and HSA_HOCl_ with the same fluorescent dye using amine and aminooxy-labeling chemistry, respectively, and analyzed their uptake by THP-1-derived M1 macrophages. The different degrees of labeling of HSA_UT_ and HSA_HOCl_ were adjusted by adding unlabeled protein at the same molar concentration to HSA_UT_ until the same fluorescence intensity per molar amount of protein as the one of labeled HSA_HOCl_ was reached. As shown in Fig. 4 a and b, cells accumulated approximately 2.5-fold more HSA_HOCl_ than HSA_UT_, supporting our hypothesis that HSA_HOCl_ may act as potent ligand of scavenger receptors and interfere with SR-mediated antigen uptake. However, while the fluorescence moiety is identical in both HSA_UT_ and HSA_HOCl_, we cannot exclude that the different modes of attachment of the fluorescent dyes have a certain influence on the cellular uptake of the proteins.

### 3.5 HOCl-modified serum albumin binds to scavenger receptor CD36

Several lines of evidence show that, among the main scavenger receptors of THP-1-derived macrophages, CD36, SR-B1 and SR-A, CD36 plays a major role in the uptake of oxidized LDL in the body [71,72]. Since this receptor specifically recognizes the oxidized form of LDL formed upon hypochlorite stress, it seems conceivable that it might also recognize and bind HOCl-modified HSA and other oxidized and chlorinated proteins. To test, if HSA_HOCl_ associates with CD36, we first incubated M1 macrophages with native HSA and HSA_HOCl_ at 0 °C to allow surface binding but not the internalization of HSA, before a fluorescent anti-CD36 antibody, which specifically binds to the ligand binding site of the receptor, was added to the cells. As shown in Fig. 4 c and d, no significant differences in the binding of anti-CD36 could be observed upon incubation with HSA_UT_, when compared to the buffer control, indicating that HSA_UT_ rather does not associate with CD36. In contrast, binding of anti-CD36 was reduced by approximately 30%, when the cells were pre-incubated with HSA_HOCl_. These results suggest that CD36 may be one SR which mediates the internalization of oxidized proteins by THP-1-derived M1 macrophages.

### 3.6 HOCl-modified serum albumin converts antigens into ligands of scavenger receptor CD36

HSA is typically present in a large molar excess over foreign antigens at sites of infection (up to 750 μM in blood, 300 μM in interstitial fluid) [55,56,73]. Due to the high HOCl concentration in chronically inflamed tissues, which can be as high as 25–50 mM [61], it could be speculated that HOCl-modified HSA is present in excess over an antigen as well. Since HOCl-modified HSA itself exhibits oxidizing and chlorinating properties [74], it is reasonable to assume that at least some antigenic molecules become modified by HSA_HOCl_. Hence, one possible explanation for the reduced OVA uptake in the presence of HSA_HOCl_ may be that modification by HOCl converts HSA into a potent ligand of CD36 which then competes with HSA_HOCl_-modified OVA for the same receptor binding site. When present in sufficiently high amounts, HSA_HOCl_ could just outcompete the modified OVA and entirely prevent its uptake via CD36. To explore this hypothesis, we first tested whether the uptake of OVA in the presence of HSA_HOCl_ could generally be increased by adding higher amounts of antigen to the cells, while keeping the HSA_HOCl_ concentration constant (Fig. 4 e). Indeed, internalization of OVA in the presence of HSA_HOCl_ increased with increasing amounts of antigen added, demonstrating that the inhibitory effect of HSA_HOCl_ on OVA uptake depends on the concentration of the antigen. This also excludes the possibility that the observed HSA_HOCl_-mediated reduction in antigen uptake is due to a general cytotoxic effect of HSA_HOCl_. Next, we pre-incubated THP-1-derived M1 macrophages with an anti-CD36 antibody to block the CD36 ligand binding site, before we added fluorescent OVA together with HSA_UT_ or HSA_HOCl_. After 2 h of incubation, the uptake of OVA by the cells was re-analyzed by flow cytometry (Fig. 4 f). Internalization of OVA by M1 macrophages that have been incubated in the absence or presence of HSA_UT_ was not affected by the blocking of the CD36 receptor, confirming that this scavenger receptor is not responsible for the uptake of non-modified native antigens. In the presence of HSA_HOCl_, however, blocking of CD36 led to a significantly decreased uptake of OVA. Strikingly, a similar effect was observed when OVA had been co-incubated with HOCl instead of HSA_HOCl_, strongly suggesting that both HOCl- and HSA_HOCl_-mediated modification of OVA leads to the redirection of the antigen to CD36 for uptake. Inspection of OVA after treatment with HOCl and HSA_HOCl_ on a reducing and non-reducing SDS gel revealed that, in contrast to HOCl, presence of HSA_HOCl_ does not lead to observable irreversible misfolding and aggregation OVA, indicating that successful recognition of modified OVA by CD36 does not depend on the formation of irreversible aggregates (Supplementary Figure S4).

Finally, to test whether HSA_HOCl_ and the modified OVA compete for the same receptor binding site, we co-incubated the cells with 50 μM HSA_HOCl_ and different amounts of fluorescent OVA that had been pre-treated with HOCl (OVA_HOCl_). Indeed, uptake of OVA_HOCl_ in the presence of HSA_HOCl_ increased with increasing amounts of the modified antigen added, strongly suggesting that HOCl- or HSA_HOCl_ -modified OVA and HSA_HOCl_ are internalized by the same scavenger receptor in our experimental setting (Fig. 4 g). CD36 therefore appears to be one receptor that specifically recognizes and binds proteins which have been modified by HOCl or its derived chloramines.

### 3.7 Conversion of serum albumin and antigens into CD36 ligands is reversible

Our results indicate that HOCl-induced modifications enable HSA to bind to the same scavenger receptors as does HOCl- and HSA_HOCl_ -modified OVA, leading to a concentration-dependent reduction of the uptake of this antigen.

Findings from our previous work strongly suggest that reversible N-chlorination of basic amino acid residues by HOCl is likely the critical modification, which mediates the different physiological effects of AOPPs, such as HOCl-treated HSA [16]. We therefore wondered, whether the observed conversion of HSA into a potent scavenger receptor ligand upon HOCl treatment might also be mediated by N-chlorination. Since protein-derived chloramines were reported to be unstable species which decompose in a time-dependent manner when incubated at temperatures above 4 °C [75], we first analyzed the decay of the HSA-derived chloramines. We determined that a fresh preparation of HSA_HOCl_ contained about 25 N-chloramines per HSA molecule using a chloramine detection assay according to Dypbukt et al. [16,45] (Supplementary Fig. S5 a). The number of chloramines present in HSA_HOCl_, decreased progressively to about 50% of the initial chloramine content after 6 h of incubation under the conditions used for our experiments. After 2 h of incubation in the presence of THP-1 cells (i.e. the total duration of the antigen uptake assays), however, HSA_HOCl_ retained at least 80% of its chloramines formed upon HOCl treatment (Supplementary Fig. S5 b).

To test, whether the conversion of HSA and OVA into CD36 ligands upon HOCl and HSA_HOCl_ -treatment, respectively, is generally reversible and thus, could be reversed by antioxidants, we added the antioxidant ascorbate directly to the cells which were incubated with OVA-488 in the presence of HSA_HOCl_ to remove reversible modifications, such as N-chlorination and thiol oxidation (e.g. sulfenylchlorides). As shown in Fig. 4 f, uptake of OVA in the presence of ascorbate was no longer mediated by CD36, since prior blocking of the CD36 receptor had no effect on the internalization of the antigen. Moreover, there was no observable shift in the migration of OVA on a non-reducing gel, when ascorbate has been added after 1 h of incubation of OVA with HSA_HOCl_, when compared to native OVA (Supplementary Fig. S4 c and d). These results strongly suggest that reversible amine chlorination and/or thiol modifications are sufficient to allow recognition and binding of proteins by CD36.

### 3.8 Inhibitory effect of HOCl-modified serum albumin on antigen uptake can be reversed by antioxidants

Since neither modified OVA nor HSA_HOCl_ are ligands of CD36 upon treatment with antioxidants, it can be expected that HSA_HOCl_ does not affect the uptake of OVA after treatment with ascorbate and other reductants. To test this, we incubated THP-1-derived M1 macrophages with fluorescent OVA in the presence of various amounts of ascorbate-reduced HSA_HOCl_ (HSA_HOCl_ + Asc), before the uptake of OVA by the variously treated cells was again analyzed by flow cytometry. Indeed, treatment of HSA_HOCl_ with ascorbate strongly diminished its inhibitory effect on the internalization of OVA-488 (Fig. 4 h, i). Presence of HSA_HOCl_ + Asc, even at the highest concentration tested, reduced antigen uptake by at most 20%. Methionine, another known reducing agent for N-chloramines showed a similar effect (Supplementary Fig. S6). Analysis of HSA_HOCl_ after treatment with the different reductants on reducing and non-reducing SDS gels did not show evidence that ascorbate or methionine was able to reduce HSA's intrinsic disulfides, whereas DTT reduction led to an observable migration shift (Supplementary Fig. S2).

These results further support the idea that reversible N-chlorination and/or thiol oxidation of HSA is most likely responsible for its increased binding affinity to scavenger receptors and hence, its inhibitory effect on the internalization of antigens by M1 macrophages.

### 3.9 Inhibitory effect of HOCl-modified serum albumin on antigen uptake depends on HOCl-induced N-chlorination of its basic amino acid side chains

Finally, we tested whether HOCl-induced N-chlorination of basic amino acid residues or the modification of HSA's single free cysteine (Cys34) is responsible for the observed inhibitory effect of HSA_HOCl_ on the uptake of OVA.

To prevent N-chlorination, we first blocked free amino groups of lysine and nitrogens in the guanidino-moiety of arginine residues in HSA via selective methylation, before we treated the now methylated HSA again with HOCl (HSA_Met_ + HOCl). Presence of HSA_Met_ + HOCl, even at the highest concentration, did not significantly reduce antigen uptake (Fig. 4 j, k). This shows that methylation of HSA prevents HOCl-based activation of the inhibitory effect. However, we cannot fully exclude that HSA's single free cysteine was modified during the methylation procedure, despite the fact that it was carried out under reductive conditions. Nevertheless, taken together with the fact that the HOCl-induced modifications are reversible with both ascorbate and methionine, these results suggest that N-chlorination of basic amino acid side chains is the main mechanism responsible for the inhibitory effect of HSA_HOCl_.

### 3.10 Intracellular processing of antigens is impaired by HOCl-modified human serum albumin

**Fig. 5:**
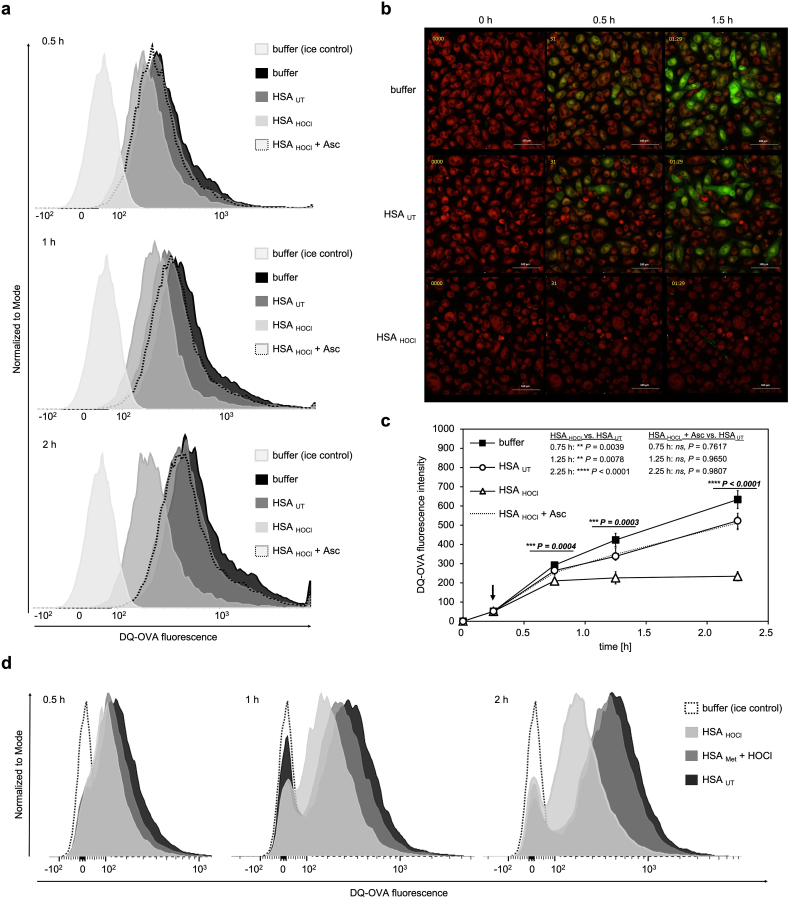
**HOCl-modified human serum albumin inhibits intracellular antigen processing.** THP-1-derived M1 macrophages were first incubated with 50 μg mL^−1^ DQ-OVA for 15 min (time point: 0 h), before HSA_UT_, HSA_HOCl_, ascorbate-treated HSA_HOCl_ (HSA_HOCl_ + Asc), methylated HSA after HOCl treatment (HSA_Met_ + HOCl) or buffer was added to the cells to a final concentration of 50 μM. After incubation for up to 2 h, intracellular processing of DQ-OVA was assessed by (**a**, **c, d**) flow cytometry and (**b**) live-cell fluorescence microscopy. **(a, d)** Single-parameter histogram overlays of DQ-OVA fluorescence of the various samples 0.5, 1 and 2 h after addition of HSA or buffer are shown. (**b**) Images of live cells processing DQ-OVA (processed DQ-OVA is visible in the green channel), which were taken before (0 h) or 0.5 and 1.5 h after addition of HSA or buffer are shown. Cells were stained with the CellTracker™ Red CMTPX Dye (red channel). **(c)** Processing of DQ-OVA is reflected by the median fluorescence intensity of the variously treated cells measured at different time points. Black arrow indicates time point of HSA/buffer addition. Means and standard deviations were derived as described in the “Material and Methods” section. One-way ANOVA test followed by a Tukey post-hoc test was performed to determine the significance of the differences between the group means at each time point. The resulting P value of the one-way ANOVA test is indicated in bold and italic above the line. The P values of the Tukey post-hoc test for the indicated pair-wise comparisons (HSA_HOCl_ vs. HSA_UT_ and HSA_HOCl_ + Asc vs HSA_UT_) are shown for each time point. P values < 0.05 indicate statistical significance; **P < 0.01; ***P < 0.001; ****P < 0.0001. ns = not significant. (For interpretation of the references to colour in this figure legend, the reader is referred to the Web version of this article.)

Next, we asked whether HOCl-modified HSA may also affect the intracellular processing of antigens already encountered. As model antigen we used BODIPY-conjugated DQ-OVA, a self-quenched conjugate of OVA that exhibits green fluorescence upon proteolytic degradation proportional to the extent of DQ-OVA processing. As we already knew from our previous experiments that HSA_HOCl_ interferes with antigen uptake, we first pulsed THP-1-derived M1 macrophages with DQ-OVA to allow for sufficient internalization, followed by washing to remove excess protein. Subsequently, native and variously treated HSA, or buffer was added to the cells and DQ-OVA degradation was monitored over a period of up to 2 h via flow cytometry or in real-time by live-cell fluorescence microscopy (Fig. 5). Processing of DQ-OVA in the absence of HSA occurred at the highest rate within the first 30 min after the 15-min pulsing period and then proceeded at a constant, albeit slightly slower rate. Cells treated with HSA_UT_ generated fluorescent degradation products with comparable kinetics. Cells incubated with HSA_HOCl_, however, exhibited a marked increase in fluorescence in the first 30 min indicative of effective antigen degradation, but no further increase in fluorescence signal could be observed after this time point, showing that HSA_HOCl_ does not only slow down, but ultimately inhibits antigen processing. Importantly, the effect of HSA_HOCl_ was completely abolished upon reduction with ascorbate (Fig. 5 a, c) or by prior methylation of its amino groups (Fig. 5 d), strongly suggesting that N-chlorination is the responsible mechanism for the functional switch of HSA into an efficient inhibitor of antigen processing as well. These findings suggest that the inability of THP-1-derived M1 macrophages to present antigens upon exposure to HSA_HOCl_, is not only attributable to lack of antigen capture, but also due to impaired processing of already internalized antigens.

### 3.11 Presence of HOCl-modified human serum albumin leads to a dose-dependent decrease in MHC class II expression

Since efficient MHC class II expression is crucial for antigen presentation, we wondered whether HOCl-modified HSA could also interfere with IFN-γ-induced MHC II expression in macrophages, providing another explanation for their lower antigen presenting capacity and the diminished CD4^+^ T cell response observed upon exposure to HSA_HOCl_.

**Fig. 6:**
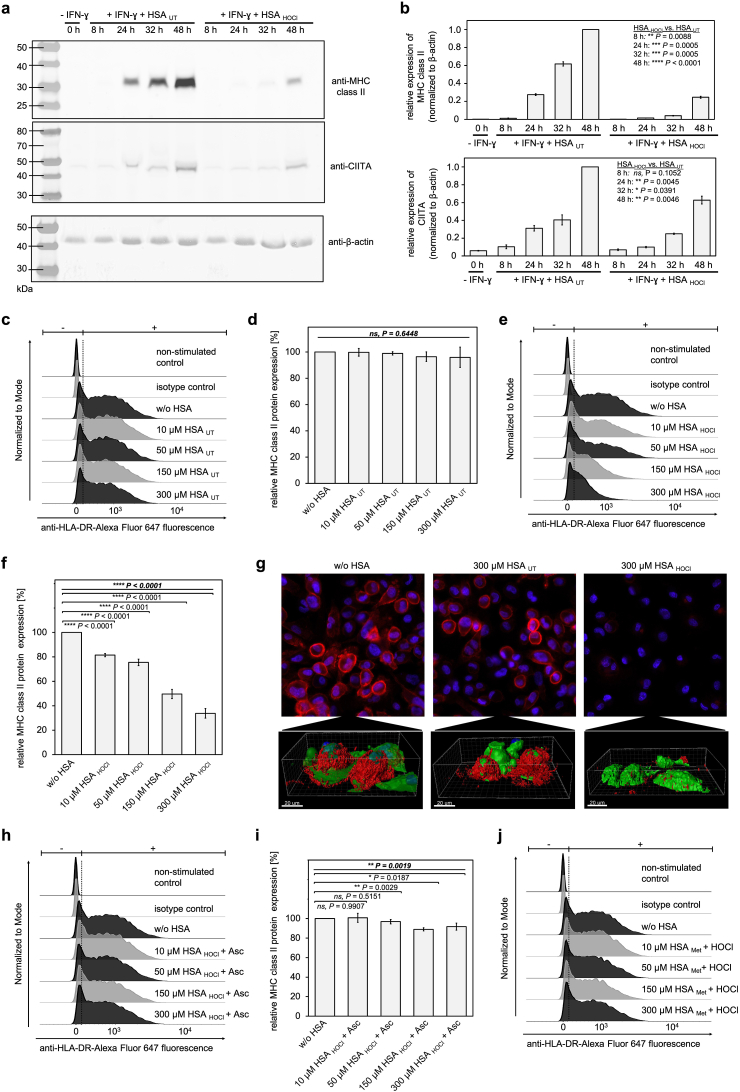
**N-chlorinated human serum albumin decreases IFN-γ-induced expression of MHC class II proteins by THP-1-derived M1 macrophages in dose-dependent manner. (a, b)** THP-1-derived M0 macrophages were incubated with 50 μM HSA_UT_ or HSA_HOCl_ together with IFN-γ for up to 48 h. Total expression of MHC class II, CIITA and β-actin was evaluated by immunoblotting using specific antibodies. The loaded volumes were adjusted such that, for each treatment condition, the same amount of protein extract (i.e. equivalent number of extracted cells) was analyzed. Relative densities of MHC class II and CIITA bands were normalized to β-actin. Relative density of sample “+IFN-γ + HSA_UT_ 48 h” was set as reference. **(a)** A representative Western blot analysis is shown. **(b)** Results of three Western Blot analyses are shown (means and standard deviations). Welch's two independent sample *t*-test for unequal variances was performed to determine the significance of the differences between HSA_HOCl_ and HSA_UT_ at each time point. P values < 0.05 indicate statistical significance; *P < 0.05; **P < 0.01; ***P < 0.001; ****P < 0.0001. ns = not significant. **(c**–**j)** Various amounts of HSA_UT_, HSA_HOCl_, ascorbate-treated HSA_HOCl_ (HSA_HOCl_ + Asc) or methylated HSA after HOCl treatment (HSA_Met_ + HOCl) were added to THP-1-derived M0 macrophages together with IFN-γ. After 6 h of incubation, level of MHC class II cell surface expression by the variously treated cells was analyzed by **(c-f, h-j)** flow cytometry and **(g)** high-resolution confocal fluorescence microscopy. **(c**, **e, h, j)** Single-parameter histogram overlays of anti-HLA-DR-Alexa Fluor 647 fluorescence in the various samples are shown. **(d**, **f, i)** Results of three independent experiments are shown (means and standard deviations). General expression of MHC class II proteins by the immune cells in the absence of HSA (w/o HSA) was set to 100%. Basal MHC II expression by non-stimulated M0 macrophages was subtracted from the fluorescence values of stimulated cells. One-way ANOVA test was performed to determine the significance of the differences between the group means in one data set. The resulting P value is indicated in bold and italic above the line at the top of each data set. If P < 0.05, a Dunnett post-hoc test was performed. The resulting P values for the pair-wise comparison of the outermost 2 bars within the individual brackets are shown. P values < 0.05 indicate statistical significance; *P < 0.05; **P < 0.01; ****P < 0.0001. ns = not significant. **(g)** Upper panel: Representative fluorescence images of the cells, that have been incubated in the presence of HSA_UT_, HSA_HOCl_ or buffer. Cell nuclei were stained with DAPI; MHC class II complexes are shown in red. Lower panel: Representative high-resolution image of 2 cells of each cell sample. Cytoplasm of the cells was stained with the CellTracker™ Green CMFDA Dye; cell nuclei with DAPI. MHC class II complexes at the cell surface are shown in red. (For interpretation of the references to colour in this figure legend, the reader is referred to the Web version of this article.)

First, to test whether HSA_HOCl_ has any effect on the IFN-γ-induced expression of MHC class II molecules, we added a low non-cytotoxic amount of HSA_HOCl_ (i.e. 50 μM) to PMA-differentiated, resting M0 macrophages together with IFN-γ and incubated the cells for up to 48 h. Total levels of MHC class II transactivator (CIITA), which plays a key role in the regulation of MHC class II gene expression [76], and MHC class II proteins were then evaluated by immunoblotting using specific antibodies. Induction of the MHC class II expression with IFN-γ in the presence of HSA_UT_ led to a steady increase of the CIITA and MHC class II protein levels over time (Fig. 6 a, b). Incubation of the cells with HSA_HOCl_, however, strongly reduced the accumulation of CIITA and MHC class II proteins. 48 h upon stimulation with IFN-γ, the protein levels of CIITA and MHC class II were respectively ~30% and 70% less in cells treated with HSA_HOCl_ than in those which were co-incubated with HSA_UT_. Notably, the amounts of β-actin, a continuously expressed and ubiquitously present protein with a mRNA half-life of approximately 14 h [77], did not decrease, even after 48 h of incubation with HSA_HOCl_, compared to HSA_UT_, indicating that HSA_HOCl_ rather does not act as a general inhibitor of protein synthesis (Supplementary Fig. S7).

Next, to test whether the effect of HSA_HOCl_ on the IFN-γ-induced expression of MHC class II molecules is dose-dependent, varying amounts of HSA_UT_ and HSA_HOCl_ were added to M0 macrophages immediately before polarization into the M1 phenotype was induced with IFN-γ. To avoid excessive cytotoxicity of higher HSA_HOCl_ concentrations, the overall incubation time in the presence of HSA did not exceed 6 h. MHC class II cell surface expression by the variously treated cells was then evaluated by flow cytometry and confocal fluorescence microscopy using fluorescently-labeled monoclonal antibodies against human MHC class II molecules (Fig. 6 c-g).

In line with expectations, induction of MHC class II expression with IFN-γ resulted in a significant amount of MHC II molecules presented at the cell surface of polarized macrophages. Presence of HSA_UT_ during M1 polarization, even at the highest concentration tested (i.e. 300 μM), did not affect MHC II expression, as the detected MHC II levels were comparable to those observed in the absence of HSA (Fig. 6 c, d, g). Treatment of the cells with HSA_HOCl_, however, strongly decreased the amount of expressed MHC II molecules (Fig. 6 e, f, g). The suppressive effect of HSA_HOCl_ on IFN-γ-stimulated MHC II expression became more pronounced with increasing amounts of HSA_HOCl_ added. Finally, almost no MHC II complexes could be observed on single-cell level, when the HSA_HOCl_ concentration reached 300 μM, i.e. the maximum reported HSA concentration in interstitial fluids (Fig. 6 g). Interestingly, this observed effect was largely abrogated after the treatment of HSA_HOCl_ with the reductant ascorbate or by prior methylation of HSA's basic amino acids, indicating that the ability of HSA_HOCl_ to inhibit MHC class II expression is most likely conferred by reversible HOCl-induced N-chlorination (Fig. 6 h-j).

These results strongly suggest that N-chlorinated HSA does not only affect antigen uptake and processing, but also leads to generally decreased levels of MHC class II proteins.

### 3.12 HOCl-modified human serum albumin does not increase the degradation rate of MHC class II proteins

**Fig. 7:**
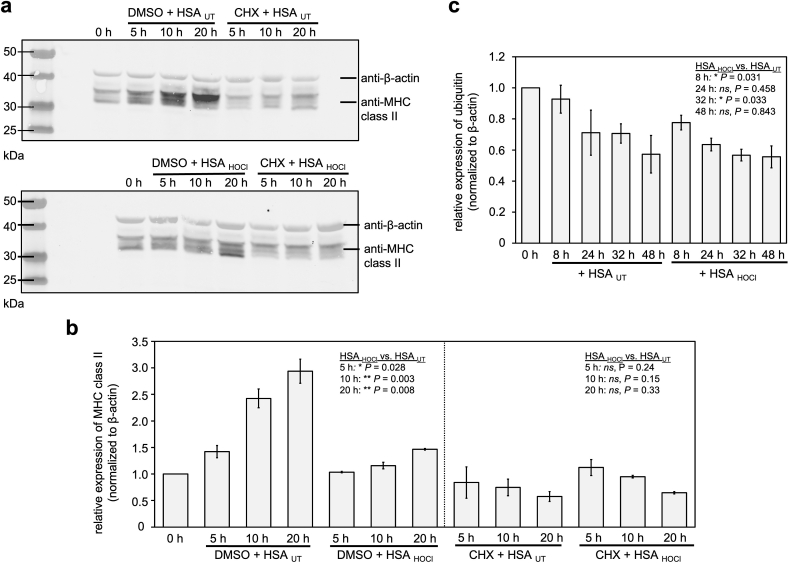
**HOCl-modified human serum albumin does not increase degradation rate of MHC class II proteins.** THP-1-derived M1 macrophages were pre-incubated with cycloheximide (CHX) or vehicle (DMSO), before 50 μM HSA_UT_ or HSA_HOCl_ were added to the cells for the indicated time periods. Total expression of MHC class II proteins or ubiquitin was evaluated by immunoblotting using specific antibodies. Relative densities of MHC class II proteins or ubiquitin were normalized to β-actin. **(a)** A representative Western blot analysis is shown. **(b, c)** Results of three Western Blot analyses are shown (means and standard deviations). Total MHC class II protein or ubiquitin amount prior to the addition of HSA (0 h) was set as reference. Welch's two independent sample *t*-test for unequal variances was performed to determine the significance of the differences between HSA_HOCl_ and HSA_UT_ at each time point. P values < 0.05 indicate statistical significance; *P < 0.05; **P < 0.01. ns = not significant.

One possible mechanism by which HSA_HOCl_ decreases the accumulation of MHC class II proteins might be through its potential interference with IFN-γ-signaling leading to a decreased expression rate of MHC class II encoding genes. Another explanation for the observed reduced accumulation of MHC class II proteins in the presence of HSA_HOCl_ may, however, also be a faster degradation of already synthesized MHC class II molecules. To test this idea, we performed a cycloheximide (CHX) chase assay to compare the stability of MHC class II proteins in the presence or absence of HSA_HOCl_. We, thus, treated THP-1 derived M1 macrophages with cycloheximide to inhibit *de novo* protein synthesis, before we added a low, non-cytotoxic amount of HSA_HOCl_ (i.e. 50 μM) or native HSA to the cells. After incubation for up to 20 h, we evaluated the total MHC class II protein levels again by immunoblotting (Fig. 7 a, b). Incubation of M1 macrophages with HSA_UT_ in the absence of CHX led to an increase in the amount of MHC class II proteins due to the presence of IFN-γ in the medium. In line with our previous findings, presence of HSA_HOCl_ strongly reduced the accumulation of MHC class II proteins compared to HSA_UT_. Upon inhibition of protein synthesis with CHX, a progressive decrease in MHC class II levels could be observed over time. Importantly, there was no difference in the MHC class II protein degradation rate between cells treated with HSA_UT_ and HSA_HOCl_, respectively, suggesting that HSA_HOCl_ rather does not act upstream of MHC class II translation. To confirm this latter conclusion, we also tested whether HSA_HOCl_ increases overall protein degradation by the ubiquitin proteasome system, which catalyzes the degradation of MHC class II proteins and most other proteins in mammalian cells [78]. As shown in Fig. 7 c, HSA_HOCl_ did not enhance the ubiquitination of proteins when compared to HSA_UT_, supporting our hypothesis that HSA_HOCl_ reduces global MHC class II protein levels rather by inhibiting IFN-γ-induced expression of MHC class II encoding genes than by enhancing the degradation of already synthesized MHC class II molecules. However, further experiments are needed to elucidate the mechanism by which HSA_HOCl_ interferes with IFN-γ signaling.

The combined results of our study demonstrate that HSA_HOCl_ is a highly potent modifier of the antigen presentation process, which targets several crucial steps simultaneously, ultimately leading to a substantial inhibition of the adaptive immune response.

## 4 Discussion

In this study, we investigated the effect of HOCl-modified HSA on the antigen presenting capacity of monocyte-derived macrophages. By performing a direct T cell activation and proliferation assay, we first showed that incubation of APCs with the antigen Tetanus toxoid (TT) in the presence of HOCl-modified HSA leads to decreased response of TT-specific CD4^+^ T cells, indicative of a disturbed MHC II-dependent antigen presentation process. Antigen presentation is complex and requires a sequence of distinct steps, including antigen internalization, antigen processing and loading of antigen peptides onto MHC II proteins for presentation - all of them could principally be affected by HOCl-modified HSA.

Other studies have likewise demonstrated that various types of chronic stress, such as long-term ethanol consumption [79,80], exhaustive physical exercises [81], or chronic viral infection [82,83] can reduce the antigen presenting ability of macrophages, but the intracellular mechanisms responsible for the suppressed antigen presentation remain largely unknown.

One mechanism that might account for the poor presentation of TT by the THP-1-derived M1 macrophages could be poor antigen capture. In our previous study, we showed that HOCl-induced N-chlorination converts HSA into a chaperone holdase which binds to and protects other proteins against protein aggregation under HOCl stress [16]. Such association of HSA_HOCl_ with antigenic proteins could potentially decrease their propensity to enter immune cells – possibly due to impaired recognition of the HSA-antigen complex by scavenger receptors - and thus, block the antigen presentation process already at the entry level. Indeed, we have previously demonstrated that HSA_HOCl_ is capable of binding the mycobacterial antigen Ag85B and preventing its uptake by neutrophil-like cells [16], and a similar phenomenon was also observed in other studies, where HSA_HOCl_ was shown to effectively bind envelope proteins of HIV and West Nile virus and prevent entry of the virus into host cells [84,85].

We found that HSA_HOCl_ decreases the uptake of the model antigen ovalbumin (OVA) in a dose-dependent manner and, when present in high, yet still physiologically relevant amounts, can even inhibit its receptor-mediated internalization. Importantly, this effect was not seen upon-re-reduction of HSA_HOCl_ with methionine or ascorbate, a mild reductant, which reduces N-chloramines and reversible thiol modifications (e.g. sulfenylation and sulfenylchlorides), or upon prior methylation of its basic amino acid residues, strongly supporting an N-chlorination based mechanism as well.

One explanation for the reduced antigen uptake in the presence of N-chlorinated HSA would be that HSA_HOCl_ competes with OVA for the binding to the same receptor. While the receptors implicated in the uptake of native and HOCl-modified OVA by dendritic cells (DCs) have been well defined [86,87], the corresponding receptors have not yet been identified in THP-1-derived macrophages.

THP-1 macrophages were found to mainly express the scavenger receptors CD36, SR-A and SR-B1 [88]. Although there is little doubt that all of these receptors can recognize and bind oxidized proteins, such as oxidized LDL, several lines of evidence suggest that SR-A and SR-B1 are significantly down-regulated in THP-1 cells upon polarization into the pro-inflammatory M1 phenotype [89,90], indicating that these receptors may not play a primary role in the uptake of oxidized proteins by THP-1-derived M1 macrophages.

In contrast, CD36 was found to be the major SR responsible for the internalization of oxidized LDL by THP-1-derived M1 macrophages [72]. Since this receptor specifically recognizes the oxidized form of LDL formed upon hypochlorite stress [71], it seems feasible that it can also recognize and bind other oxidized and chlorinated proteins. Indeed, we found that, in contrast to HSA_UT_, HSA_HOCl_ significantly associates with CD36.

Based on the fact that HSA-derived chloramines have oxidizing and chlorinating properties [74], HSA_HOCl_ may convert native OVA into a ligand of CD36 and thus, compete with the now modified OVA for binding to the same receptor binding site. Indeed, we found that blocking of CD36 clearly reduced the uptake of OVA in the presence of HSA_HOCl_, but not native HSA, and similar observations were made when HOCl instead of HSA_HOCl_ was added to the cells. Importantly, internalization of OVA in the presence of HOCl or HSA_HOCl_ was no longer mediated by the CD36 receptor upon addition of ascorbate, suggesting that reversible chlorination of basic amino acid residues and/or thiol modifications are sufficient to allow recognition and binding of the modified OVA by CD36. Interestingly, a similar phenomenon was reported in studies using dendritic cells, where binding of native OVA to the mannose receptor was shown to be rather inefficient, but its affinity to another receptor, Lox-1, could be strongly increased upon treatment with HOCl [87]. One may, thus, speculate that exposure to HOCl transforms not only HSA but also other proteins in its immediate vicinity into high affinity ligands of particular scavenger receptors. Since HSA is typically present in high molar excess over foreign antigens at sites of infection [55,56,73] and likely becomes modified by HOCl, HSA_HOCl_ could just outcompete its less abundant counterparts, providing one possible explanation for the dose-dependent reduction of antigen uptake in the presence of HOCl-modified HSA.

Since monocytes are able to acquire antigens before they extravasate into tissues and retain them for presentation much later in their life cycle, after appropriate maturation [91,92], we asked whether HSA_HOCl_ could also influence intracellular processing of already internalized antigens. Here we found that even low amounts of N-chlorinated HSA could strongly inhibit intracellular degradation of OVA. Accumulating evidence suggests that the processing pathogenic antigens requires the autophagic machinery. For example, the mycobacterial antigen Ag85B was shown to be intracellularly processed for MHC II presentation via autophagy *in vivo* [93]. Autophagy can be suppressed by activation of PI3K/Akt [114], which is one of the effects of HSA_HOCl_ [16].

Monocytes acquire MHC class II expression progressively during differentiation into macrophages [76]. In our model system, the presence of HSA_HOCl_ during the IFN-γ-induced M1 polarization reduced the MHC class II protein level in a dose-dependent manner. Because HSA_HOCl_ had no significant effect on the degradation kinetics of MHC class II proteins, it seems highly likely that HOCl-modified HSA exhibits its effect downstream of protein synthesis i.e. by interfering with IFN-γ-induced expression of MHC class II encoding genes. Recent studies imply a role of PI3K/Akt signaling in this process as well. Chandrasekaran et al. found that pharmacologic activation of PI3K signaling can disrupt the cellular response to IFN-γ and inhibit expression of MHC class II by decreasing STAT1 expression [94]. Indeed, we found that the presence of HSA_HOCl_ did not only decrease the synthesis rate of MHC class II proteins, but also that of CIITA, prompting us to speculate that HSA_HOCl_ controls MHC class II gene expression through activation of the PI3K/Akt signaling pathway as well [16].

In summary, we demonstrate that macrophages lose their ability to efficiently present newly acquired antigens via MHC class II molecules upon their encounter with increased levels of HOCl-modified HSA – a condition which commonly occurs within inflamed tissues. This HSA_HOCl_-induced impairment in antigen presentation is not exclusively due to poor antigen internalization and intracellular antigen degradation but is also caused by down-modulation of MHC II expression, pointing towards a global suppressive effect of HSA_HOCl_ on this process. Especially the latter mechanisms could prevent antigen presentation in an environment hostile to cells of the adaptive immune response. However, since defective antigen presentation can prolong disease by slowing down the immune system's adaptive response at an initial stage, our findings support a role of HOCl-modified HSA and potentially also other AOPPs in the development and the progression of chronic infection and inflammation.

## Declaration of competing interest

None.
