# Supplementary material for: Hypochlorous acid-modified human serum albumin suppresses MHC class II - dependent antigen presentation in pro-inflammatory macrophages
Source: Redox Biol. 2021 Apr 20;43:101981. doi: 10.1016/j.redox.2021.101981 (PMC8105673; doi:10.1016/j.redox.2021.101981)
Supplement: Multimedia component 1 [file mmc1.docx]

**SUPPLEMENTARY DATA**

**
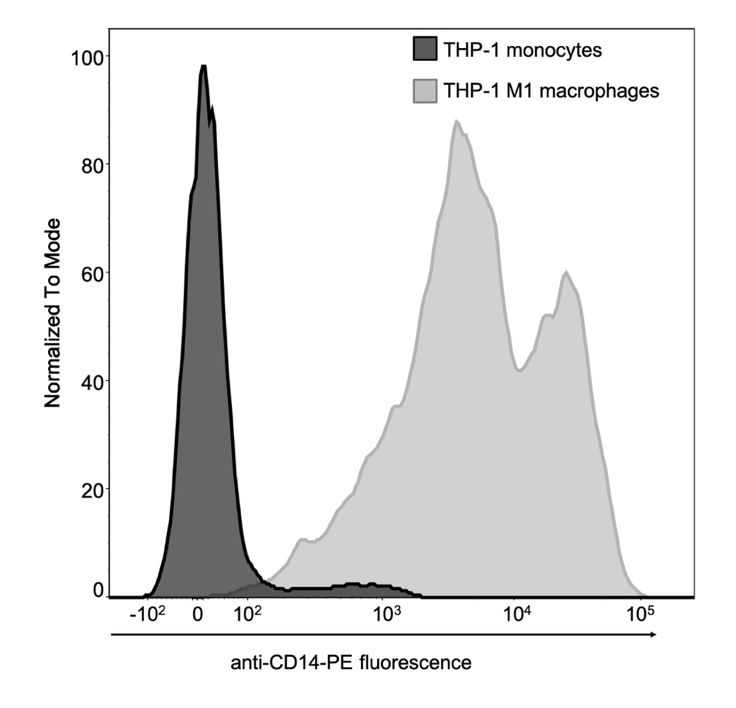
**

**Supplementary Figure S1: Expression of CD14 on THP-1 monocytes and THP-1-derived M1 macrophages.** For immunofluorescent staining, monoclonal PE-labeled anti-CD14 antibodies were used. Representative data in CD14 expression on THP-1-derived M1 macrophages (light grey) compared with undifferentiated THP-1 monocytes (dark grey) are shown.


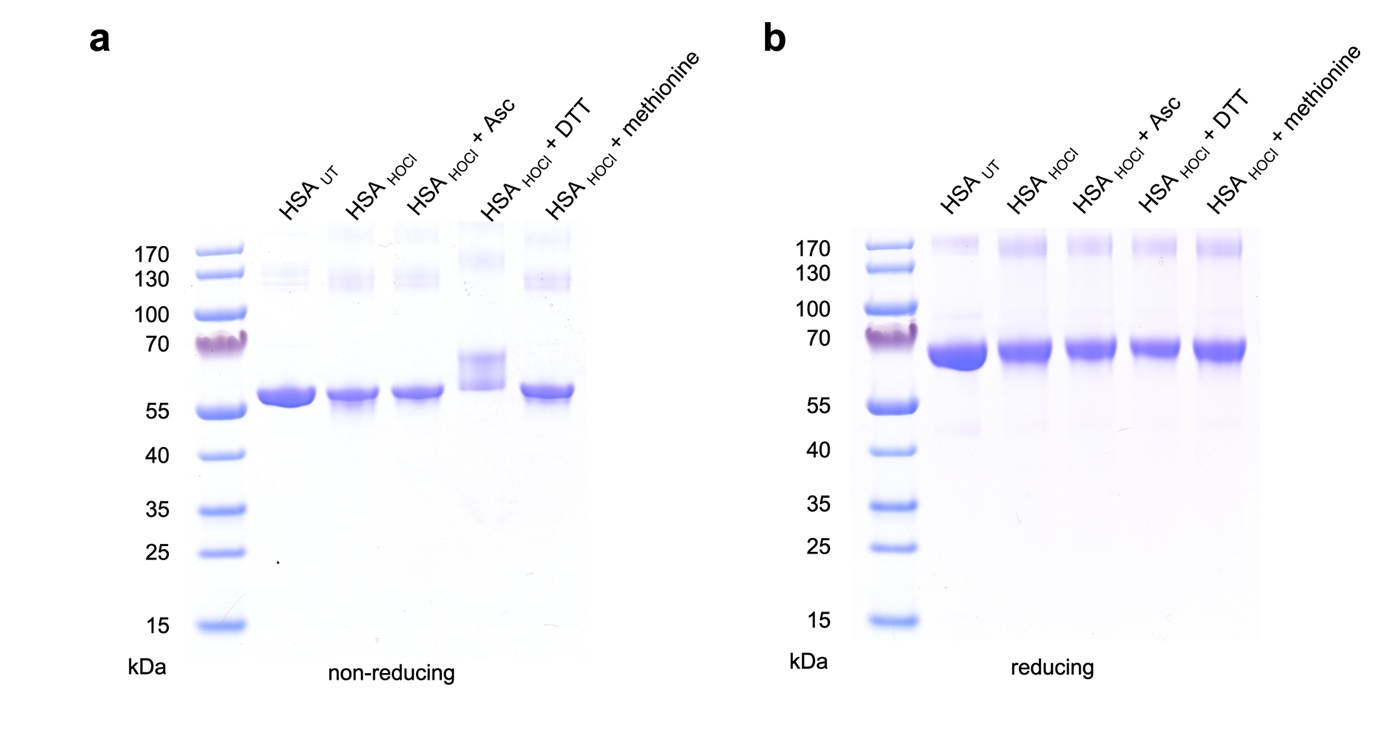


### Supplementary Figure S2: Influence of HOCl and reducing agents on the migration of human serum albumin on reducing and non-reducing SDS-PAGE gels, HSA, untreated or treated with a 50-fold molar excess of HOCl and subsequently reduced with various reductants separated on a (a) non-reducing and (b) reducing SDS gel. HOCl-treatment of HSA does not lead to appreciable fragmentation or accumulation of disulfide linked dimers, that could potentially form by oxidation of the single free cysteine in HSA. DTT-treatment leads to a change in the migration of HSA, presumably by reducing intramolecular disulfide bonds. Neither ascorbate nor methionine induce a change in migration. On a reducing gel (containing β-mercaptoethanol in the loading buffer) all samples migrate to the same, compared to the non-reducing gel, higher position.

###
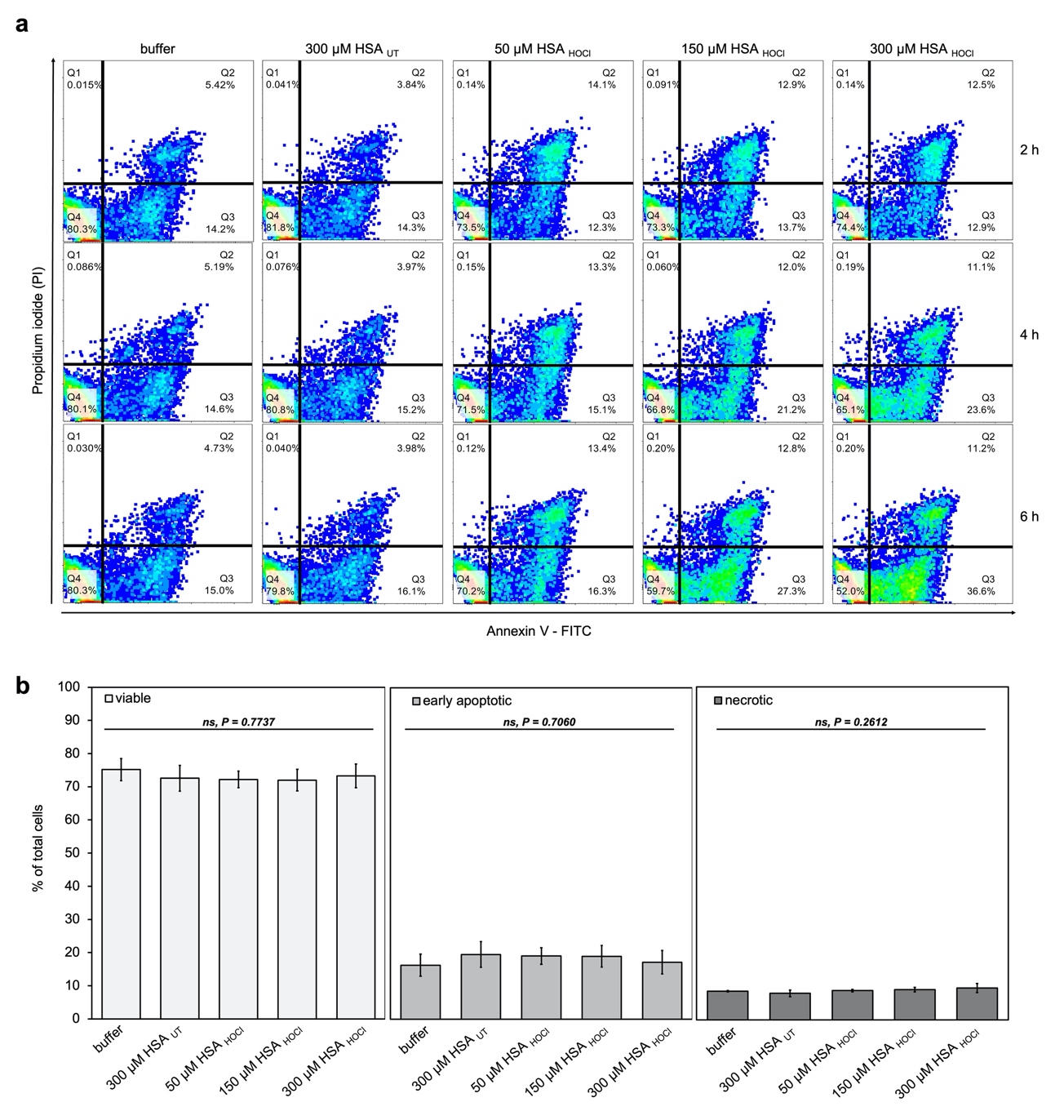


**Supplementary Figure S3: Exposure to HOCl-modified HSA led to a dose- and time-dependent decrease of the ratio of viable to early apoptotic cells. (a)** After incubation of THP-1 monocytes with 300 μM native HSA, 50-300 μM HOCl-treated HSA or buffer for two, four, or six hours, viability of the variously treated cells was evaluated by flow cytometry using Annexin V-FITC/propidium iodide (PI) staining. 30,000 events were acquired and recorded per sample. Data were analyzed using FlowJo (version 10) software. Results shown are representative of three experiments. Data plots were generated from analysis of ungated data. Viable cells (Annexin V and PI negative) appear in the lower left quadrant (Q4), early apoptotic cells (Annexin V positive; PI negative) in lower right quadrant (Q3) and late apoptotic/necrotic cells (Annexin V and PI positive) in the upper right (Q2) quadrant. **(b)** THP-1 monocytes exhibited a reduced viability of 75-80%, an average apoptosis rate of 18% and a necrosis rate of up to 8% at the beginning of the experiment (time point “0”). Results of three experiments are shown (means and standard deviations). One-way ANOVA test was performed to determine the significance of the differences between the group means in one data set. The resulting P value is indicated in bold and italic above the line at the top of each data set. P values < 0.05 indicate statistical significance. ns = not significant.

###
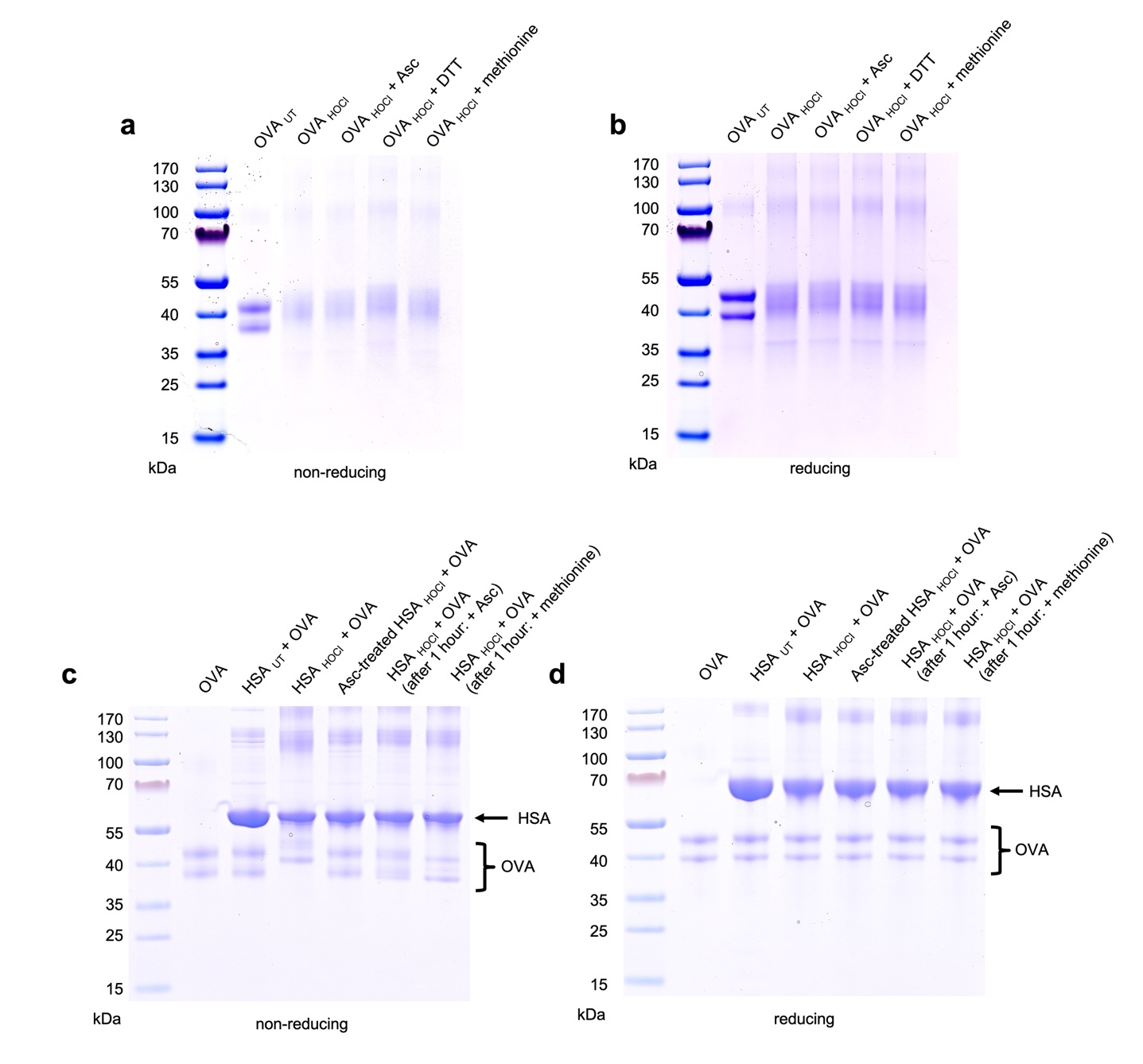


### Supplementary Figure S4: Influence of a treatment with HOCl and HOCl-modified serum albumin on the migration of ovalbumin on reducing and non-reducing SDS-PAGE gels. (a, b) OVA, untreated or treated with a 50-fold molar excess of HOCl and subsequently reduced with various reductants separated on a reducing and non-reducing gel. HOCl-treatment of OVA leads to structural changes in OVA which cannot be reversed by any of the reductants tested. (c, d) Untreated HSA and ascorbate-treated HSA_HOCl_ have no effect on the migration of OVA. Treatment with HSA_HOCl_ leads to a change in the migration of OVA, which cannot be seen on a reducing gel or upon addition of ascorbate or methionine to OVA, which has been pre-incubated with HSA_HOCl_ for 1 hour.

**
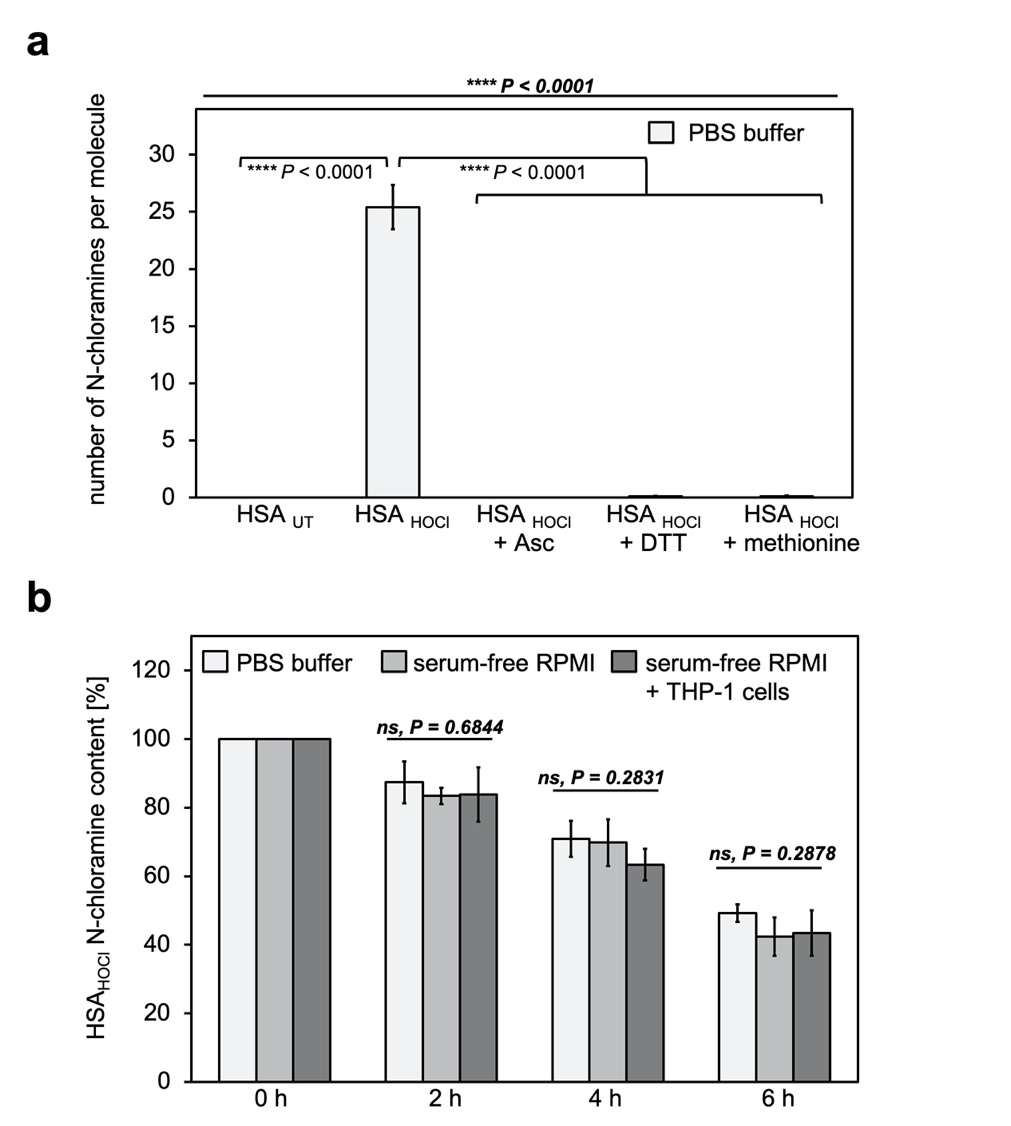
**

**Supplementary Figure S5: Chloramine content of human serum albumin after HOCl-treatment. (a)** Quantified chloramine content of native HSA, HSA_HOCl_ and HSA_HOCl_ after treatment with different reductants in 1x PBS buffer, pH 7.4. For quantification, a standard curve generated with known quantities of taurine N-chloramine was used. One-way ANOVA test followed by a Tukey post-hoc test was performed to determine the significance of the differences between the group means in the data set. The resulting P value of the one-way ANOVA test is indicated in bold and italic above the line at the top of the diagram. The resulting P values of the post-hoc test for the indicated comparisons are shown. **(b)** Decay of chloramines of HSA_HOCl_. HSA_HOCl_ was incubated at 30 °C in 1x PBS buffer, pH 7.4 and serum-free RPMI 1640 medium in the absence or presence of THP-1 cells. The chloramine concentrations were assayed at the times indicated. Results of three experiments are shown (means and standard deviations). One-way ANOVA test was performed to determine the significance of the differences between the group means for each time point. The resulting P value is indicated in bold and italic above the line at the top of each data set. P values < 0.05 indicate statistical significance; **** P < 0.0001. ns = not significant.

###
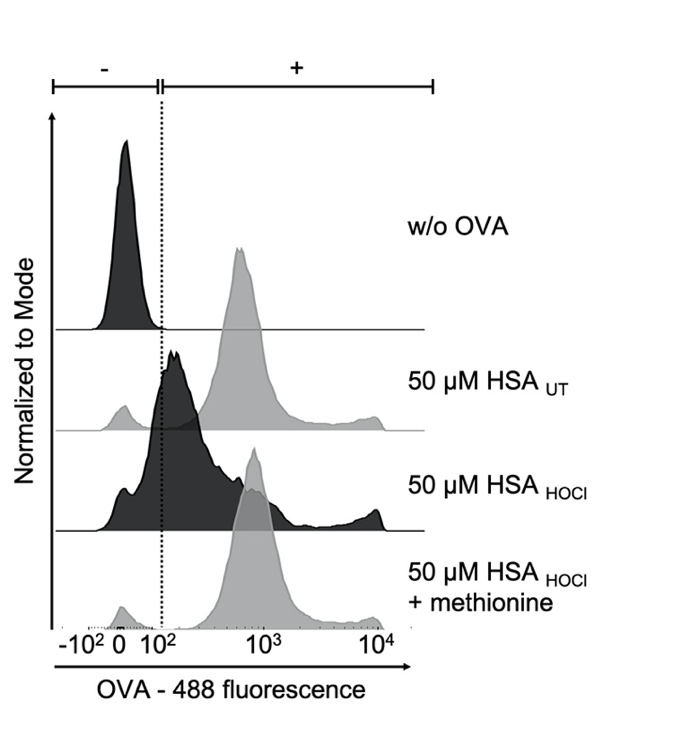


### Supplementary Figure S6: Inhibitory effect of HOCl-modified human serum albumin on antigen uptake is abrogated upon reduction with methionine. THP-1-derived M1 macrophages were incubated with 50 μM HSA_UT_, HSA_HOCl_ and methionine-treated HSA_HOCl_ (HSA_HOCl_ + methionine) together with OVA-488 for 2 hours, before OVA uptake was analyzed by flow cytometry. A representative histogram of OVA-488 fluorescence in the variously treated cells is shown.

*
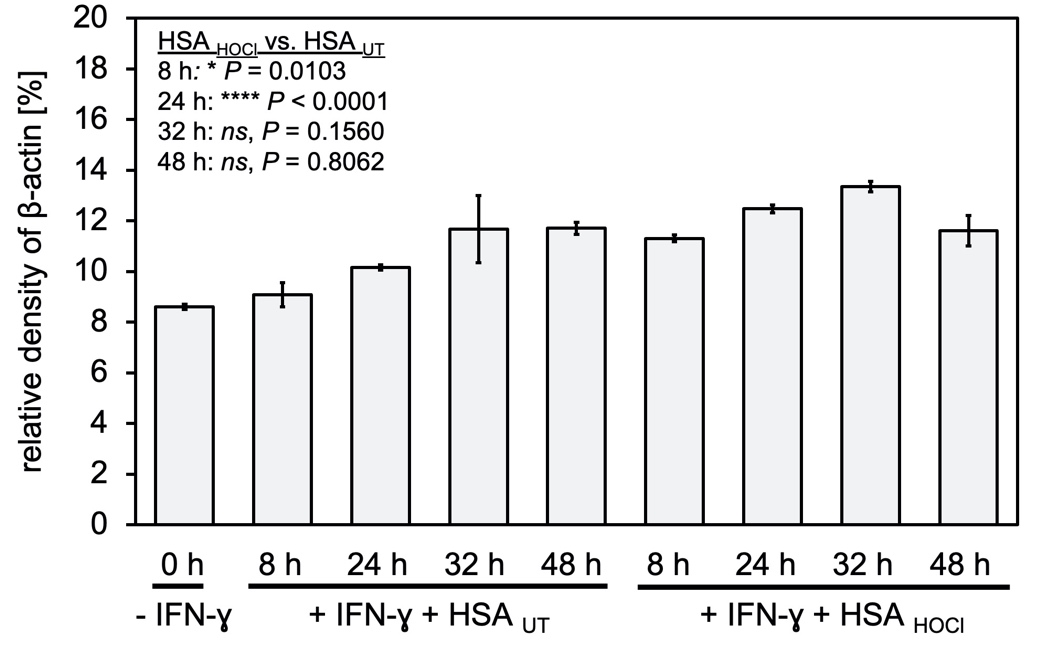
*

**Supplementary Figure S7: HOCl-modified human serum albumin does not act as a general inhibitor of protein synthesis.** THP-1-derived M0 macrophages were incubated with 50 μM native HSA (HSA_UT_) or HOCl-treated HSA (HSA_HOCl_) together with IFN-γ for up to 48 hours. Total expression of β-actin was evaluated by immunoblotting using specific antibodies. The loaded volumes were adjusted such that, for each treatment condition, the same amount of protein extract (i.e. equivalent number of extracted cells) was analyzed. Results of three Western Blot analyses are shown (means and standard deviations). Welch’s two independent sample t-test for unequal variances was performed to determine the significance of the differences between HSA_HOCl_ and HSA_UT_ at each time point. P values < 0.05 indicate statistical significance; * P < 0.05; **** P < 0.0001. ns = not significant.
